# Antibacterial efficacy and mechanism of the novel antimicrobial peptide lachnospirin-1 against *Acinetobacter baumannii*

**DOI:** 10.1080/21505594.2026.2646808

**Published:** 2026-03-16

**Authors:** Pengfei She, Mengna Li, Yiqing Liu, Guanqing Huang, Shaowei Guo, Dan Xiao, Yelan Hong, Lihua Lu, Yong Wu

**Affiliations:** a Department of Laboratory Medicine, The Third Xiangya Hosipital, Central South University, Changsha, China; b Department of Laboratory Medicine, Xiangya Hosipital, Central South University, Changsha, China; c Department of Laboratory Medicine, Changsha First Hospital (Affiliated Changsha Hospital of Xiangya School of Medicine Central South University ), Changsha, China; d Department of Laboratory Medicine, Postgraduate Cooperative Training Base of Changsha First Hospital, Hengyang Medical College, University of South China, Changsha, China

**Keywords:** Antimicrobial peptide, acinetobacter baumannii, carbapenem-resistance, cell membrane, lipopolysaccharide, oxidative stress

## Abstract

The treatment of carbapenem-resistant *A. baumannii* (CRAB) infections has become a major global medical challenge; therefore, there is an urgent need for new antimicrobial development. Antimicrobial peptides hold great promising potential due to their rapid bactericidal activity and low induction of drug resistance. In this study, we screened and synthesized the antimicrobial peptide lachnospirin-1, which exhibited significant bactericidal activity against CRAB and can also effectively eliminate its biofilms and persister cells. Mechanism studies, including fluorescent probe detection, transmission electron microscopy observations, and molecular dynamics simulations, et al. indicated that lachnospirin-1 exerts its effects through multiple mechanisms, such as disrupting bacterial cell membranes, neutralizing lipopolysaccharide, as well as inducing oxidative stress, which also demonstrates its certain potential. In addition, the favorable safety profile and effective *in vivo* bactericidal activity of lachnospirin-1 were determined by mouse models. Overall, our study found that lachnospirin-1 has considerable research potential as a lead compound and possesses latent value as a clinical therapy for refractory infections caused by CRAB.

## Introduction

*Acinetobacter baumannii* is an opportunistic pathogenic Gram-negative bacillus and one of the main pathogens causing hospital-acquired infections. It can cause varied types of infection, including meningitis, respiratory tract infections, urinary tract infections, and sepsis [1]. The emergence of its multidrug-resistant strains has led to widespread and persistent hospital-acquired infections globally [2]. With the abuse of antibiotics, the incidence of multidrug-resistant *Acinetobacter baumannii* (MDR-AB) has been increasing, which poses a serious threat to global public health security. In 2024, CRAB has been listed as a critical priority pathogen by the World Health Organization, attracting the attention of the global medical community [3]. Compared with carbapenem-susceptible *A. baumannii*, CRAB is associated with a higher risk of mortality and morbidity [4]. CRAB is one of the five pathogens with the highest mortality rates from antibiotic-resistant infections worldwide [5]. It is estimated that in Southeast Asia, East Asia, and Oceania, its mortality rate ranks first [6]. At present, few drugs are available for the effective treatment of such bacterial infections, making it difficult to address the unmet clinical needs [7]. This underscores the ongoing challenges in this field and the urgent need for investment in research and development.

Biofilms are a crucial factor contributing to bacterial drug resistance, enabling bacteria to adhere to biotic or abiotic surfaces and to survive in harsh environments [8]. *A. baumannii* exhibits stronger biofilm-forming ability than many other bacteria, which facilitates the development of antibiotic resistance [8,9]. In hospitals, *A. baumannii* often adheres to and forms biofilms on medical devices, leading to widespread nosocomial infections [10]. CRAB exhibits resistance to a wide range of antibiotics, and the complexity of clinical environments further increases the frequency of biofilm formation. Therefore, therapeutic options for CRAB infections are extremely limited, and exploring strategies capable of eradicating CRAB and its biofilms is urgently needed.

Antimicrobial peptides (AMPs) are a class of polypeptides widely present in animals, plants, and even microorganisms, typically consisting of 20–60 amino acid residues [11,12]. Most AMPs kill bacteria through multiple mechanisms, such as acting on bacterial cell membranes or intracellular targets [13]. AMPs hold broad prospects as potential therapeutic agents and have been clinically applied as antiviral drugs (e.g. Telaprevir [14]). AMPs with immunomodulatory properties are currently under clinical trials [15], while peptide drugs targeting yeast and bacterial infections (e.g. LL-37 and PAC-11 [16]) are also in the research and development stage. Although most AMPs exhibit broad-spectrum activity, some are only effective against closely related members of the same species or genus [17]. Such AMPs are more targeted than the traditional broad-spectrum antibiotics [18,19]. In addition, unlike conventional antibiotics, many AMPs have a low rate of resistance evolution and do not show cross-resistance with other widely used antibiotic classes [20].

Due to the structural instability of natural AMPs, artificial intelligence (AI) has become a transformative tool for the design and synthesis of AMPs [21]. Therefore, by reviewing the literature and searching AMP databases, we identified a synthetic AMP lachnospirin-1 with excellent antibacterial potential. As reported by Santos-Júnior et al. [22], they employed machine learning techniques to predict and classify global microbiome AMPs curated in current public databases, and screened 100 cyclic antimicrobial peptides (cAMPs) from the AMPSphere database. Among these peptides, four exhibited a minimum inhibitory concentration (MIC) as low as 1 µM, namely cagicin-1, cagicin-4, enterococcin-1 (active against *A. baumannii*), and cagicin-1 and lachnospirin-1 (active against vancomycin-resistant *E. faecalis*), which was comparable to the MIC values of some potent peptides reported in the literature. Nevertheless, their work was limited to superficial evaluations of antibacterial activity and the establishment of simple wound models, lacking systematic in-depth investigations and mechanistic exploration. In this study, we conducted in-depth investigations into the *in vitro* and *in vivo* antibacterial activity of lachnospirin-1, and explored its potential mechanisms of action and molecular targets for the first time. The antibacterial activity was evaluated via comprehensive and multilevel antibacterial phenotypic assays both *in vitro* and *in vivo*, while the mechanism of action was elucidated by combining fluorescence assays, molecular docking, and molecular dynamics simulations, which enhanced the scientific rigor of this study.

Through the series of experiments mentioned above, we aim to verify the potent antibacterial activity of lachnospirin-1 against CRAB, which exerts bactericidal effects by targeting bacterial cell membranes via lipopolysaccharide binding and inducing oxidative stress. Meanwhile, we intend to investigate the *in vivo* antibacterial efficacy of the antimicrobial peptide and conduct a preliminary toxicity evaluation. Overall, this study provides a viable alternative therapy for the clinical treatment of CRAB infections.

## Materials and methods

### Chemicals, strains, and culture conditions

*A. baumannii* strain ATCC 19,606, *Klebsiella pneumoniae* ATCC 700,603, and *Escherichia coli* ATCC 25,922 were purchased from the American Type Culture Collection (ATCC, USA). The multidrug-resistant clinical isolates AB1069 and AB1280 were kindly provided by Chen Cha (Guangdong Provincial Hospital of Chinese Medicine, Guangzhou, China). Vancomycin-Intermediate *Staphylococcus aureus* SAJ1 was collected at the Third Xiangya Hospital of Central South University (Changsha, China) [23]. *Pseudomonas aeruginosa* PAO1 was provided by Qiao Minqiang (College of Life Sciences, Nankai University, Tianjin, China). Clinical strain *E. faecium* VSE1 was separated from the urine of patients in the Clinical Laboratory of Third Xiangya Hospital from 2016 to 2021. All clinical isolates were identified using a Vitek-2 compactsystem (bioMérieux, France) and a matrix-assisted laser desorption ionization-time of flight mass spectrometer (Bruker Daltonics, Bremen, Germany). All strains were stored in a 30% (v/v) glycerol solution at −80°C. Gram-negative bacilli were cultured in Luria-Bertani (LB) broth (Solarbio, Shanghai, China) at 37°C. Staphylococci and enterococci were cultured in tryptic soy broth (TSB) (Solarbio, Shanghai, China) and brain heart infusion (BHI) broth (Solarbio, Shanghai, China), respectively. The sequences of the antimicrobial peptides enterococcin-1, ampspherin-4, and lachnospirin-1 were SIKLIKTVKVVEKIIVEFLKKIKKFSVI, VIKKATMIDFSLKEIMLMLIKKILTSR, and LKQLNRFKYKIIKIKRIIKL, respectively, and all the peptides were synthesized by GL Biochem Ltd. (Shanghai, China). Ampspherin-4 and lachnospirin-1 were solubilized in double-distilled water, whereas enterococcin-1 was solubilized in dimethyl sulfoxide (DMSO), with all samples stored at 4°C under neutral conditions. All antibiotics used in this study were purchased from MedChemExpress (New Jersey, USA).

### Minimal inhibitory concentration (MIC) determination

The broth microdilution method was employed to determine the antimicrobial activity of the antimicrobial peptide. First, peptide diluted in water was added to untreated flat-bottom polystyrene 96-well plates at a twofold serial dilution gradient ranging from 64 to 1 μM. Subsequently, the peptide was exposed to a bacterial inoculum of 2 × 10^6^ CFU/mL suspended in LB medium (for pathogenic bacteria) and BHI broth (for intestinal commensal bacteria), respectively. After incubation, the absorbance value of each well was measured at a wavelength of 630 nm using a spectrophotometer. Three biological replicates were set up for the experiment to ensure statistical reliability [22,24].

### Short-term bactericidal activity determination

*A. baumannii* was cultured overnight in LB broth, diluted to 1.5 × 10^6^ CFU/mL with 1× PBS (pH = 7.4), and then mixed with indicated concentrations of LL-20. The colony-forming units (CFUs) were counted at 0 h, 5 min, 10 min, 30 min, 1 h, 2 h, and 4 h. The half lethal concentration (LC_5__0_) was calculated based on the CFU counts at the time point of 5 min [25].

### Time−killing assay

The mid-log phase of *A. baumannii* was diluted with MH broth containing 2-fold diluted antibiotics to obtain a final concentration of 1 × 10^6^ CFU/mL. The bacterial suspension was incubated at 37°C at 180 rpm, and 10 μL of the suspension was serially diluted and inoculated on sheep blood agar at 0, 1, 2, 4, 8, 12, and 24 h for viable cell counting. After the incubation at 37°C for 24 h, the CFUs were calculated [26].

### Biofilm eradication assay

Overnight-cultured *A. baumannii* was diluted in 1:100 with MH broth and 100 μL of the bacterial suspension was added to each well in a 96-well plate. The plate was incubated at 37°C for 24 h to form biofilms. After biofilm formation, the planktonic cells were removed by 1× PBS washing, and indicated concentrations of lachnospirin-1 were added to each well. After a static incubation at 37°C for 24 h, the biofilms were washed with saline three times to remove planktonic cells. For crystal violet (CV) staining, after air drying, the biofilms were stained with CV (0.25% wt/vol) for 15 min at room temperature. Then, the biofilms were washed with saline to remove unbound dye, and 100 μL 95% ethanol was added to dissolve the dye for 20 min. The absorbance at 570 nm (*A*_570_) was measured by a spectrophotometer (Bio-Rad, USA). For XTT staining, following air-drying, 100 μL of freshly prepared XTT staining solution was added; the samples were incubated at 37°C for 2 h, after which the absorbance was determined directly at a wavelength of 490 nm with a microplate reader [27]. Polymyxin B (PolyB) (16 μg/mL) was used as the positive control.

### Postantibiotic effect (PAE)

Bacteria in the logarithmic growth phase were washed twice with 1× PBS and resuspended to a concentration of 1.5 × 10^6^ CFU/mL. Subsequently, lachnospirin-1 at the specified concentration was added for treatment, followed by incubation at 37°C with shaking at 180 rpm for 1 h. After diluting 1000-fold with MH broth, colony counting was performed at the time points of 0, 2, 4, 6, 12, and 24 h [28].

### Hemolysis assay

Human red blood cells were washed three times with 1× PBS and resuspended to a concentration of 10% (v/v). Subsequently, the cells were mixed with equal volumes of lachnospirin-1 solutions at indicated concentrations and incubated statically at 37°C for 1 h. Triton X-100 (0.1%, v/v) and double-distilled water (ddH_2_O) were used as positive and negative control, respectively. After the incubation, 100 µL of the supernatant was transferred to a 96-well plate, and the absorbance (*A*) was measured at the wavelength of 570 nm. The hemolysis rate was calculated using the following formula [29]:

Hemolysis (%) = $\frac{A_{\mathrm{sample}}-A_{\mathrm{ddH}20}}{A_{0.1\%\mathrm{TritonX}-100}-A_{\mathrm{ddH}}20}$ ×100%.

### Persistent cell killing assay

*A. baumannii* AB1069 was cultured overnight in LB broth. Then, carbonyl cyanide m-chlorophenylhydrazone (CCCP) was added into the suspension to the final concentration of 200 μg/mL [30]. After the incubation at 37°C for 3 h, the bacterial pellets were collected by centrifugation at 3000 rpm for 10 min, washed and resuspended in 1× PBS to the final concentration of 1 × 10^8^ CFU/mL. Subsequently, the persistent cells were treated with antimicrobials at specified concentrations. At the time point of 0, 2, 4, and 6 h, the total number of viable persistent cells was calculated, respectively, by using the plate counting method.

### Persister cell killing assay

*A. baumannii* was cultured in an LB medium at 37°C with shaking at 180 rpm for 24 h to the stationary phase to obtain persister cells. The cells were washed three times and resuspended with 1× PBS to the final concentration of 3 × 10^7^ CFU/mL. Then, the persister cells were treated with lachnospirin-1 at the specified concentrations, and bacterial colony counts were performed at the time point of 0, 1, 2, 3, and 4 h, respectively [31].

### Confocal laser scanning microscope (CLSM)

The overnight-cultured bacteria were diluted at a ratio of 1:100 with LB medium, and 2 mL of the bacterial suspension was transferred to a 6-well cell culture plate with a coverslip in each well. After the incubation at 37°C for 24 h, the coverslips were gently rinsed twice with saline, and the adhered biofilms were treated with indicated concentrations of lachnospirin-1. After incubation at 37°C for 24 h, the coverslips were washed with 1× PBS and stained with 10 µM SYTO9/PI in the dark for 15 min. Subsequently, the excess dye was washed off, and the biofilms were observed using a laser scanning confocal microscope (Zeiss LSM900, Jena, Germany). The excitation wavelengths of SYTO9 and PI are 485 nm and 530 nm, respectively, and the emission wavelengths are 485 nm and 630 nm, respectively, [32].

### Outer membrane permeability assay

The hydrophobic fluorescent probe 1-N-phenylnaphthylamine (NPN) uptake assay was performed to determine the outer membrane disruption by antimicrobials [33]. Briefly, *A. baumannii* AB1069 was cultured overnight in LB broth, and the cells were collected by centrifugation at 3000 rpm for 10 min. Then, the cells were washed and suspended with a HEPES buffer (5 mmol/L, pH 7.4, 150 mmol/L sodium chloride) to the OD_6__30_ of 0.5. Subsequently, 10 μM of NPN was added to the bacterial suspension and incubated with serially 2-fold diluted lachnospirin-1 for 15 min. Triton X-100 (0.1%, v/v) was used as the positive control. The fluorescence intensity at the excitation/emission wavelengths of 350 nm/420 nm was detected by a microplate reader (PerkinElmer EnVision, USA).

### PI staining

After washing the overnight-incubated *A. baumannii* twice, resuspend it in 5 mmol/L HEPES sodium buffer to a concentration of OD_630_ = 0.2. PI fluorescent dye (MedChem Express, New Jersey, USA) was added to the bacterial suspension to a final concentration of 10 μM, followed by incubation at room temperature for 30 min in the dark. Fifty microliters of the bacterial suspension were added to a black 96-well plate, then mixed with serially diluted lachnospirin-1 solutions. Use a microplate reader to measure the fluorescence intensity every 30 s for 5 min, with the excitation/emission wavelengths of 535 nm/617 nm, respectively [34]. PolyB (16 μg/mL) was used as the positive control in the assay.

### Inner membrane permeability assay

Bacteria in the logarithmic growth phase were washed and resuspended in 5 mmol/L HEPES sodium buffer to an OD_630_ value of 0.2. SYTOX Green fluorescent dye (MedChem Express, New Jersey, USA) was added to the bacterial suspension to a final concentration of 5 μM, followed by incubation at room temperature for 30 min in the dark. Fifty microliters of the bacterial suspension were added to a black 96-well plate and mixed with serially diluted lachnospirin-1. The fluorescence intensity was measured every 30 s for 5 min with the microplate reader at excitation/emission wavelengths of 485 nm/525 nm, respectively [35]. Melittin (16 μg/mL) and ddH_2_O were used as the positive control and negative control, respectively.

### Transmembrane potential detection by DiSC3(5) probe

*A. baumannii* in the mid-logarithmic growth phase was adjusted to an OD_6__30_ of 0.2 in the HEPES buffer. DiSC3 (5) (MedChem Express, New Jersey, USA) was added to the bacterial suspension to a final concentration of 2 μM. After incubation in the dark for 30 min, the bacteria were added with lachnospirin-1 at indicated concentrations, and the fluorescence intensity was measured at the excitation/emission wavelengths of 622 nm and 670 nm, respectively [26]. Melittin (16 μg/mL) and ddH_2_O were used as the positive control and negative control, respectively.

### Laurdan staining

The overnight-cultured bacterial solution was diluted at a ratio of 1:1000 with LB and further cultured to the OD_6__30_ of 1.0. Then, 10 μM Laurdan dye (MedChem Express, New Jersey, USA) was added to the suspension. After incubation in the dark for 10 min, the bacterial suspension was washed with 1× PBS, and mixed with an equal volume of lachnospirin-1. After incubation at room temperature for 30 min, the fluorescence intensity was measured with excitation and emission wavelengths of 350 nm and 435 nm/490 nm, respectively. The membrane fluidity was quantified by the Laurdan generalized polarization (GP) index [36]:

$$\mathrm{GP}=\frac{A_{435nm}-A_{490nm}}{A_{435nm}+A_{490nm}}$$

### Fluorescence labeled lachnospirin-1 tracing

*A. baumannii* AB1069 in the logarithmic growth phase was resuspended in 1 × PBS to an OD_6__30_ of 0.2. FITC-lachnospirin-1 (GL Biochem Ltd., Shanghai, China) was added to the final concentration of 16 μM, and the mixture was incubated at 37°C for 1–2 h. Twenty minutes before sampling, 20 μg/mL of Hoechst 33,342 dye (MedChem Express, New Jersey, USA) was added, and 2 min before sampling, 20 μM FM4-64 fluorescent dye (MedChem Express, New Jersey, USA) was added. The excess dye was removed by centrifugation, and the cells were resuspended in 25–50 μL 1× PBS. An aliquot of the cell suspension was spotted onto a coverslip and observed using the Zeiss LSM900 confocal microscope. The fluorescent images were acquired and processed by using ZEN Blue Edition software [37].

### All-atom molecular dynamics simulations

Molecular dynamics simulations were performed using the CHARMM36 software according to our previous report. A lipid bilayer model was constructed by using 1,2-dioleoyl-sn-glycero-3-phosphocholine (DOPC) and 1,2-dioleoyl-sn-glycero-3-phospho-(1’-rac-glycerol) (DOPG) at a ratio of 7:3. Additionally, 1-palmitoyl-2-oleoyl-sn-glycero-3- phosphocholine (POPC) and cholesterol at a 7:3 ratio were used to simulate the mammalian lipid bilayer structure [38]. The CHARMM36 force field [39] was used to describe the antimicrobial peptides, DOPC, DOPG, POPC, and Cholesterol molecules, while water was modeled using the TIP3P [40] model. The pdb2gmx module of GROMACS 2023.3 was employed to generate force field parameter files for antimicrobial peptides, DOPC, DOPG, POPC, and Cholesterol molecules. Electrostatic interactions were handled using the particle-mesh Ewald method [41]. The time step of the production simulation was set to 2 fs, and trajectories were saved every 10 ps. Trajectory snapshots were acquired via VMD [42]. Periodic boundary conditions were applied to all simulations, covering the x, y, and z directions. Five hundred nanoseconds of equilibrium simulations and molecular dynamics calculations were performed using Gromacs 2023.3 software. The final results were visualized using Gromacs and VMD programs. All simulations were independently repeated three times.

### Isothermal titration calorimetry (ITC)

The lipopolysaccharide (LPS) used in the experiment was isolated from the Escherichia coli O55:B5 and purchased from MedChem Express. First, the Nano-ITC instrument (TA-USA) needed to be cleaned. The injection needle and sample introduction needle should be rinsed 10 times each with Decon90 and ddH_2_O respectively, then placed in the card slot for later use. Use the cleaning pump to click the “Clean” program for cleaning; the cleaning process was 14% Decon90 followed by ddH_2_O. Set the titration parameters as follows: a rotation speed of 300 rpm, a titration interval of 200 s, 16 injections, temperature of 25°C (3 µL per injection). Use the titration needle to aspirate 52 µL of lachnospirin-1, and use the sample introduction needle to take 300 µL of LPS and inject it into the sample cell; add 300 µL of ddH_2_O to the reference cell. After the titration needle was loaded, enter the sample concentration in the “Control” interface and clicked “Start” to begin the titration. The data were analyzed using NanoAnalyze Data Analysis–Version 3.12.5 [43].

### Phospholipid competition assay

Varied concentrations of lachnospirin-1 were incubated with indicated concentrations of phospholipid molecules (POPC, POPG, cardiolipin (CL)) and LPS at 37°C for 15 min. Then, the mixtures were co-incubated with 1.5 × 10^6^ CFU/mL of overnight-cultured bacterial suspensions. The viable bacterial counts were determined at the time points of 0 min, 5 min, 10 min, 30 min, 1 h, and 3 h, respectively, to generate time–kill curves.

### Reactive oxygen species (ROS) quantification

*A. baumannii* strains in the mid-logarithmic phase were adjusted to OD_6__30_ of 0.5 with 1 × PBS. Subsequently, the suspension was incubated with 10 µM DCFH-DA (MedChem Express, New Jersey, USA) in the dark for 30 min. Then, 90 µL of the labeled bacterial suspension was mixed with 10 µL of lachnospirin-1 at the indicated concentrations and added to a black 96-well plate. After 30 min of incubation, the fluorescence intensity was measured by using a microplate reader at an excitation wavelength of 488 nm and an emission wavelength of 525 nm [44].

### Quantification of ROS components

The ROS mainly consists of superoxide (•O^2−^), hydrogen peroxide (H_2_O_2_), and hydroxyl radicals (•OH), which can be detected by using the fluorescent probes of HKSOX-1, HKperox-2, and HKOH-1 r (MedChem Express, New Jersey, USA), respectively [45–47]. Briefly, the bacterial suspension in the logarithmic growth phase was adjusted to the OD_6__30_ of 0.5 in 1× PBS containing 10 µM of the specific probes. After incubation in the dark for 30 min, the bacterial suspension was added with indicated concentrations of antimicrobials and incubated for another 30 min. PolyB (16 µg/mL) and ddH_2_O were used as positive and negative controls, respectively. The fluorescence intensity was monitored at the excitation/emission wavelengths of 500/520 nm (HKSOX-1), 520/543 nm (HKperox-2), and 500/520 nm (HKOH-1 r), respectively.

### Antibacterial efficacy of lachnospirin-1 in the presence of ROS scavengers

To investigate the effect of ROS scavenger on the antibacterial activity of lachnospirin-1, the MIC of lachnospirin-1 against *A. baumannii* AB1069 was determined in the presence of 5 mM reduced l-glutathione (a ROS scavenger) [48] and thiourea [49], respectively.

### Intracellular ATP quantification

The intracellular ATP concentration was detected by using an enhanced ATP assay kit (Beyotime, Shanghai, China), following the manufacturer’s instructions. Briefly, overnight-cultured *A. baumannii* AB1069 was centrifuged, washed, and resuspended with 1× PBS to an OD_6__30_ of 0.5. The bacterial suspension was then incubated with a 2-fold serially diluted lachnospirin-1, 1× PBS was used as a negative control. After the incubation at 37 °C for 1 h, the suspension was centrifugated at 10,000 rpm for 5 min at 4°C, and the precipitate was lysed using a lysis buffer. The lysate was centrifuged again, and the supernatant was mixed with the working reagent. After an incubation at room temperature for 5 min, the luminescence intensity was measured with the microplate reader.

### Stability determination

In the stability determination assay, lachnospirin-1 was treated with human serum or various inorganic salt solutions (300 mM sodium chloride, 9 mM potassium chloride, 4 mM calcium chloride, 2 mM magnesium chloride, 16 μM zinc chloride, 12 μM ammonium chloride, or 8 μM iron chloride) at 37°C for 2 h, followed by LC_5__0_ detection as described above [50]. The serum used in the experiment was pure serum from healthy adult males aged 25–35 years, obtained from the Physical Examination Center of the First Hospital of Changsha, without heat inactivation.

### Transmission electron microscopy (TEM)

Log-phased *A. baumannii* AB1069 suspension was centrifuged at 4000 rpm for 8 min, washed and resuspended in saline containing 5 mM lachnospirin-1. After incubation at 37°C with shaking at 180 rpm for 1 h, the bacteria were centrifuged, washed with 1× PBS again, and fixed with 2.5% glutaraldehyde. For the TEM sample preparation, staining was performed with 2% uranyl acetate and 2% osmium tetroxide in the dark, followed by gradient ethanol treatment and embedding in epoxy resin. The resin sections were placed on copper mesh grids and stained with lead citrate [51]. Finally, the ultrastructure of the bacterial cells was observed by using a TEM (Hitachi, Tokyo, Japan).

### Mouse subcutaneous abscess model

The study followed the ARRIVE guidelines. All animal-related procedures were approved by the Ethics Committee of the Third Xiangya Hospital of Central South University (NO. CSU-2024–0280). Female ICR mice aged 6–8 weeks, weighing 23–27 g, were purchased from Hunan SJA Laboratory Animal Co., Ltd. All mice were euthanized by cervical dislocation prior to undergoing tissue collection and other subsequent procedures. The mice were randomized over different groups using online random sequence generation at www.random.org.

After anesthetizing with 2% isoflurane in oxygen for 15 min, the hair on the back of the mice was shaved by using a razor and chemical depilatory. Log-phased cultures of *A. baumannii* AB1069 were washed and resuspended in sterile saline. Then, 100 μL of the bacterial suspension containing 1 × 10^8^ CFU/mL was subcutaneously injected into the back of the mice. One hour after infection, the mice (*n* = 18) were randomly divided into 3 groups with 6 mice per group and received subcutaneous injections of 20 mg/kg of lachnospirin-1, 20 mg/kg of PolyB, or ddH_2_O, respectively. The mice were euthanized at 24 h after treatment, and the abscess tissues were excised, homogenized in 1 mL saline, and subjected to CFU counting. Meanwhile, the abscess samples were fixed in 4% paraformaldehyde overnight. After a gradient of alcohol dehydration and xylene rehydration, the samples were embedded in paraffin, sectioned, dewaxed, and stained with hematoxylin and eosin (H&E) [52].

### Mouse wound infection model

Mice were anesthetized by the inhalation of 2% isoflurane. After shaving the hair on the back, as described above, a diameter of 6 mm wound was created. Then, 50 μL of *A. baumannii* AB1069 suspension containing 1 × 10^8^ CFU/mL cells was inoculated onto the wound surface. Two hours after the infection, moisturizing cream (Glaxal Base, Canada) containing 2% (w/w) lachnospirin-1 was applied with 2% (w/w) ddH_2_O and 2% (w/w) PolyB as the negative and control, respectively (*n* = 6/group). Each wound was covered with an impermeable membrane to prevent ointment leakage and bacterial infection. Membrane with ointment was replaced every 6 h. Mice were euthanized 48 h post infection, and the wound tissues were homogenized with saline. Bacterial counts were determined by serial dilution plating. Meanwhile, the intact wound samples were fixed in 4% paraformaldehyde for H&E staining and cytokines (IL-6, IL-1β, and TNF-α) detection, respectively [53].

### In vivo toxicity

The *in vivo* toxicity was assessed using 6–8-week-old female ICR mice via intraperitoneal injection. Mice were randomly divided into 2 groups (*n* = 6/group): the vehicle group and the 20 mg/kg lachnospirin-1 treatment group. After 24 h of the treatment, whole blood and serum samples were collected. Hematological tests were performed using a Bio-Rad BC-5000vet automatic hematology analyzer (Mindray, Shenzhen), and biochemical biomarkers were measured using a Hitachi Labospect 003 automatic biochemical analyzer (Japan). Meanwhile, the heart, liver, spleen, lung, and kidney tissues were dissected and stained with H&E for pathological observation [54].

### Statistical analysis

Data are presented as mean ± standard deviation (SD) from at least three independent experiments. All data were analyzed using GraphPad Prism 10 software. Student’s t-test was used for comparisons between two groups, while one-way analysis of variance (ANOVA) combined with Tukey’s test or Dunn’s multiple comparison test was used for comparisons among multiple groups. The statistical significance criteria were set as: *, *p* < 0.05; **, *p* < 0.01; ***, *p* < 0.001; ****, *p* < 0.0001.

## Results

### Effective antimicrobial efficacy of lachnospirin-1 against CRAB and its biofilms

**Figure 1.**
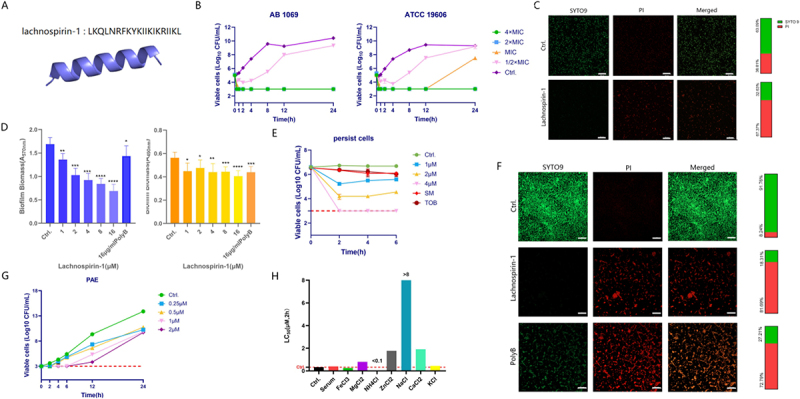
*In v-itro* antibacterial effects of lachnospirin-1. (A) Structure and sequence of lachnospirin-1. (B) Bactericidal activity of lachnospirin-1 against *A. baumannii* determined by CFU count. (C) Live/dead bacterial staining. Scale: 20 µm. (D) Quantification of biofilms by CV and XTT reduction assay. (E) Antibacterial activity of lachnospirin-1 against persister bacteria. Tob: Tobramycin; SM: streptomycin. (F) Anti-biofilm effect under confocal microscopy. Scale: 20 µm. (G) Post-antibiotic effect of lachnospirin-1. (H) Stability of lachnospirin-1 in serum and inorganic salts.

**Figure 2.**
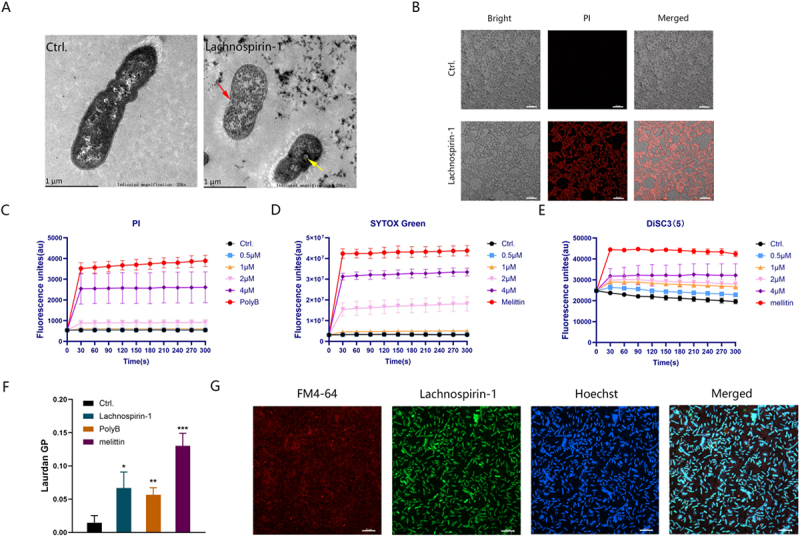
Lachnospirin-1 acts on the cell membrane of AB1069. (A) Observation of the effect of lachnospirin-1 on the cell membrane under TEM. Scale: 1 µm. Red arrows indicate cell membrane rupture and content leakage; yellow arrows indicate the appearance of mesosomes. (B) Observation of PI staining fluorescence intensity under CLSM. Scale: 20 µm. (C) Quantitative analysis of PI staining. (D) Quantitative analysis of SYTOX GREEN staining. (E) Detection of membrane potential using DiSC3(5) probe. (F) Detection of membrane fluidity by Luardan staining. (G) Fluorescence labeling of FITC-lachnospirin-1 observed under CLSM, showing its localization in the cytoplasm of *A. baumannii*. Green fluorescence represents FITC-lachnospirin-1; red fluorescence represents the membrane stain FM4-64; blue fluorescence represents the DNA-stained Hoechst 33,342. Scale: 10 µm.

**Figure 3.**
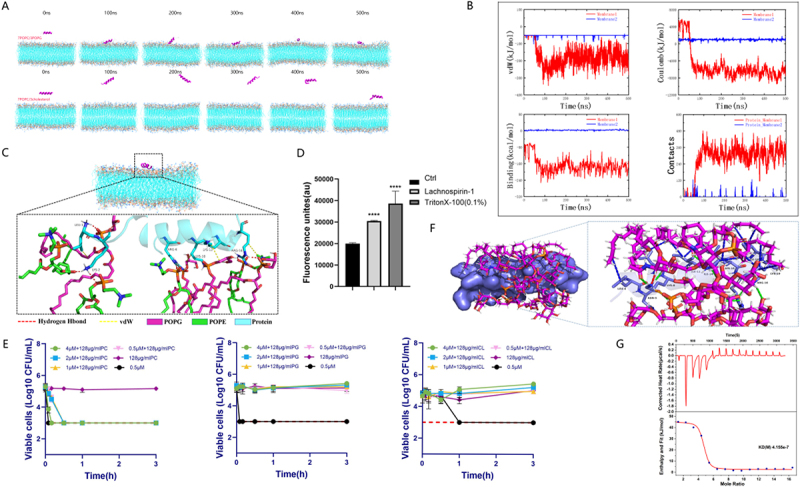
Lachnospirin-1 can specifically bind to LPS to target bacterial cell membrane. (A) Molecular dynamics simulation diagrams of lachnospirin-1 interacting with cell membranes. The upper diagram shows bacterial membranes, and the lower diagram shows mammalian membranes. From left to right: simulation initiation, membrane attachment, membrane penetration, and the equilibrium state of lachnospirin-1 interacting with the cell membrane. (B) Energy analysis during molecular dynamics simulation. The upper left panel shows the van der Waals interaction energy between the antimicrobial peptide and the cell membrane; the upper right panel shows the electrostatic interaction energy (coulomb) between the antimicrobial peptide and the cell membrane; the lower left panel shows the binding free energy between the antimicrobial peptide and the cell membrane; the lower right panel shows the binding free energy contribution of amino acid residues of the antimicrobial peptide in the mixed cell membrane system of DOPC and DOPG. Membrane1 refers to the mixed cell membrane system of DOPC and DOPG; Membrane2 refers to the mixed cell membrane system of POPC and Cholesterol. (C) Details of the interaction between the amp and the mixed cell membrane of DOPC and DOPG during molecular dynamics simulation. (D) Detection of bacterial cell membrane permeability after lachnospirin-1 treatment using NPN probe. (E) Competitive inhibition experiment of lachnospirin-1 with cell membrane components POPC, POPG, and cl. (F) Schematic diagram of the interaction structure between LPS and lachnospirin-1. Blue sticks represent lachnospirin-1 residues; magenta sticks represent LPS molecules; green dashed lines represent hydrogen bond interactions; light green dashed lines represent C-H bond interactions; red dashed lines represent electrostatic interactions; orange dashed lines represent salt bridge interactions; blue dashed lines represent hydrophobic interactions. (G) Detection of the binding affinity between lachnospirin-1 and LPS by ITC.

**Figure 4.**
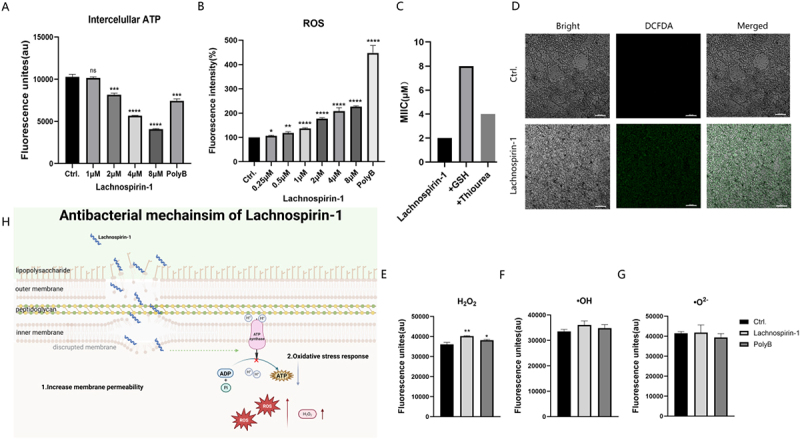
Lachnospirin-1 induces oxidative stress and stimulates the accumulation of ROS. (A) Quantitative results of intracellular ATP after treatment with lachnospirin-1. (B) Detection of intracellular ROS levels using DCFDA probe. (C) Effect of reduced GSH and thiourea on the antibacterial activity of lachnospirin-1. (D) Observation of DCF fluorescence intensity after lachnospirin-1 treatment under confocal microscopy. Scale:20 µm. (E-G) Determination of ROS component of H_2_O_2_ (E), •OH (F), •O^2-^ (G) by probes HKperox-2, HKOH-1 r, and HKSOX-1, respectively. (H) Dual antibacterial mechanisms of lachnospirin-1. ADP, adenosine diphosphate; ATP, adenosine triphosphate; ROS, reactive oxygen species.*, *p* < 0.05; **, *p* < 0.01; ***, *p* < 0.001; ****, *p* < 0.0001; ns, no statistical significance.

By searching the AMP database (sourced from AMPSpere, https://ampsphere.big-data-biology.org/), the sequences of three AMPs, lachnospirin-1, ampspherin-4, and enterococcin-1, were obtained and synthesized. Further screening was conducted using MIC determination and red blood cell hemolysis assays. Among the AMPs, lachnospirin-1 showed the most effective bactericidal activity with extremely low toxicity to RBCs, which was ultimately selected for subsequent studies. The clinical strain 1069 (AB1069), characterized by both carbapenem resistance and multidrug-resistant phenotypes, was selected as the test strain for subsequent experiments (Table S1). As shown in Figure 1(B), lachnospirin-1 exhibited significant antibacterial activity against AB1069 and ATCC19606. Meanwhile, lachnospirin-1 exhibited extremely low erythrocyte toxicity, which was far lower than its effective bactericidal concentration (Figure 2). In contrast, although enterococcin-1 had good antibacterial efficacy (Table S2), it showed high toxicity (Figure 1(B)); whereas ampspherin-4, despite its low toxicity (Figure 1(A)), had poor antibacterial activity (Table S2). Next, the antimicrobial spectrum of lachnospirin-1 was determined by calculating the LC_5__0_ and MIC. As shown in Table 1, lachnospirin-1 exhibited significant bactericidal activity against *A. baumannii* and *P. aeruginosa* with LC_50_ of 0.1–1.8 µM, the MIC values of lachnospirin-1 against *A. baumannii* ranged from 2 to 32 µM, and it also exhibited favorable antibacterial activity against some strains of *E. faecium*, *K. pneumoniae*, *P. aeruginosa*, *E. coli,* and *S. aureus*. Meanwhile, lachnospirin-1 exhibited rapid bactericidal activity, within minutes to hours (Figure 3). We have also determined the antibacterial activity of melittin against AB1069 and found that its LC_50_ was approximately 1 µM (Figure 4). Lachnospirin-1 exhibited comparable antibacterial activity to melittin. The live/dead bacterial staining further confirmed the rapid bactericidal activity by lachnospirin-1 against AB1069 (Figure 1(C)).

**Table 1.** Antibacterial susceptibility of lachnospirin-1 against pathogens.

	LC_50_(µM)	MIC (µM)	MIC (µg/mL)	
Strain	Lachnospirin-1	Lachnospirin-1	PolyB	VAN
*A. baumannii*				
ATCC 19,606	1.8	2	2	—
ATCC 1195	—	2	2	—
AB 1069*^a^	0.7	2	4	—
AB 1280*^a^	0.6	8	4	—
AB 1085	0.1	2	2	—
AB 1	—	4	4	—
AB 2	—	4	4	—
AB 3	—	2	4	—
AB 4	—	32	4	—
AB 5	—	4	2	—
*K. pneumoniae*				
ATCC 700,603	>4	>64	2	—
ATCC 13,883	—	8	2	—
*E. faecium*				
VSE1	>4	4	—	2
*P. aeruginosa*				
PAO1	0.5	4	2	—
PA 47	—	32	2	—
*E. coli*				
ATCC 25,922	>4	4	4	—
*S. aureus*				
ATCC 43,300	—	>64	—	1
SAJ1	>4	1	—	8

*, MRD-AB; ^a^, CRAB; —, no date.

**Figure 5.**
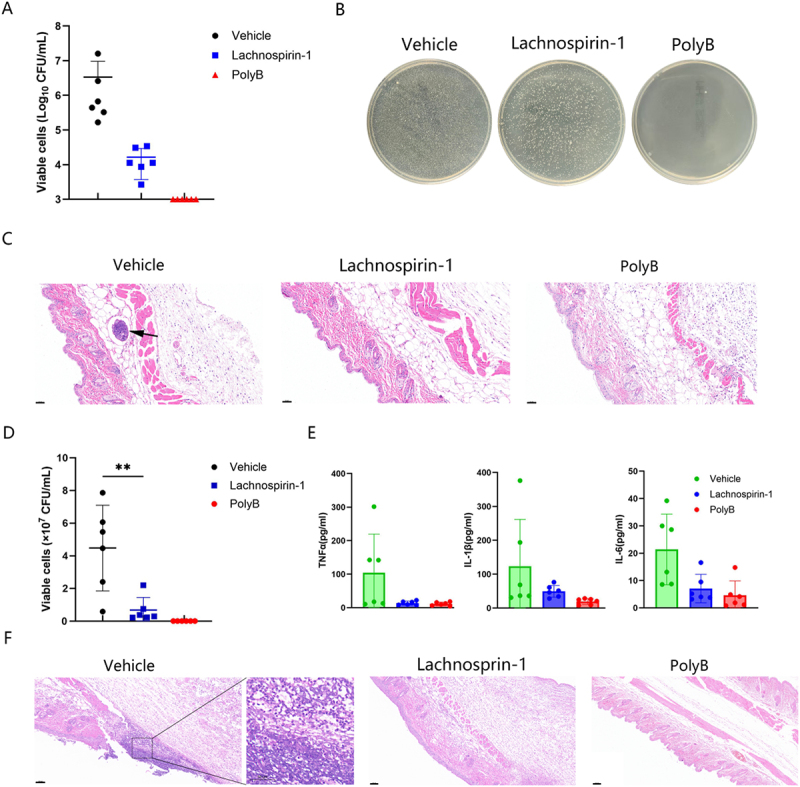
Antibacterial effects of lachnospirin-1 in mouse models. (A) Bacterial load in the skin abscess model after lachnospirin-1 treatment. (B) Observing the antibacterial effect of lachnospirin-1 via plate colony counting. (C) H&E staining of the abscess area after lachnospirin-1 treatment. Black arrows indicate abscesses. Scale: 50 µm. (D) Viable bacterial loads in wounds. (E) Inflammatory factor levels quantification among groups. (F) H&E staining of the infected wounds. Scale: 100 µm. The enlarged part shows the inflammatory infiltration area, scale: 50 µm. **, *p* < 0.01.

In clinical settings, *A. baumannii* often forms biofilms or persister cells, which greatly increase the difficulty of treatment. By CV and XTT reduction staining, lachnospirin-1 was found to exhibit effective biofilm eradication efficacy at a concentration of 1 µM, exhibiting superior biofilm clearance ability compared with PolyB group (Figure 1(D)). In accordance, CLSM observations revealed that the amount of biofilm in the lachnospirin-1-treated group was significantly reduced compared to the control group (Figure 1(F)). Compared with conventional antibiotics of streptomycin and tobramycin, lachnospirin-1 also exhibited more significant bactericidal activity against *A. baumannii* persister cells (Figure 1(E)). And lachnospirin-1 also had certain antibacterial effects on other species (Figure 5). In addition, lachnospirin-1 also showed favorable PAE against *A. baumannii* (Figure 1(G)).

**Figure 6.**
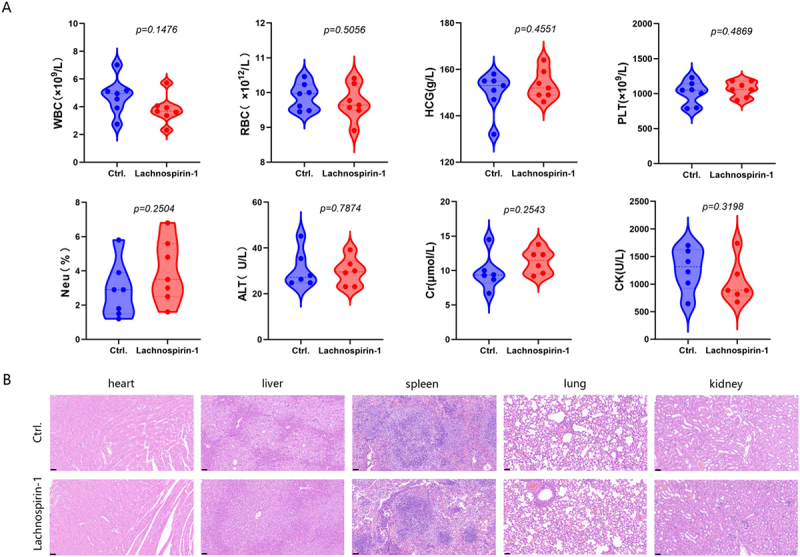
*In vivo* toxicity of lachnospirin-1. (A) Comparison of the levels of liver and kidney function markers between the two groups. (B) H&E staining of main organs. Scale: 50 µm.

Lachnospirin-1 has a single-chain helical structure (Figure 1(A)), and we determined its stability in serum and inorganic salts. The LC_50_ of lachnospirin-1 in serum was not significantly affected, in contrast, the antibacterial activity was significantly impaired in sodium chloride alone (Figure 1(H)). Notably, ammonium chloride exerted a synergistic antimicrobial effect with lachnospirin-1 (Figure 6).

### Lachnospirin-1 disrupts the cell membrane and specifically binds to lipopolysaccharide

Using TEM, we observed that the cell membranes of *A. baumannii* treated with lachnospirin-1 showed obvious damage, with leakage of contents and formation of cavity structures (Figure 2(A)). As shown in Figure 2(C), we found that the fluorescence intensity of the PI probe after lachnospirin-1 treatment increased with the increased concentration. Similarly, by CLSM observation, the red fluorescence (PI) after lachnospirin-1 treatment can also be observed (Figure 2(B)). Consistent with our expectation, the fluorescence intensity of the membrane-specific probe SYTOX Green increased in the presence of lachnospirin-1 within 5 min (Figure 2(D)). And the fluorescence intensity of DiSC3 (5) increased in a concentration-dependent manner, indicating that lachnospirin-1 depolarized the bacterial cell membrane (Figure 2(E)). Lachnospirin-1 can reduce the fluidity of the bacterial cell membrane and increase its fragility as determined by the Laurdan probe (Figure 2(F)). To further verify the destructive effect of lachnospirin-1 on the cell membrane, a fluorescent group conjugated with FITC was linked to lachnospirin-1 (FITC-lachnospirin-1). Firstly, we confirmed that addition of FITC group exhibited no impact on the antibacterial activity of lachnospirin-1 (Figure S7). By CLSM observation, green fluorescence (FITC-lachnospirin-1) was observed throughout the bacteria, blue fluorescence (Hoechst) appeared in the middle of the bacteria, and red fluorescence (FM-64) was mainly distributed in the peripheral region of the bacteria after the treatment of lachnospirin-1. Thus, we inferred that lachnospirin-1 disrupts the cell membrane, enters the bacterial interior, and exerts its antibacterial effect (Figure 2(G)).

To further explore the molecular basis of the interaction between lachnospirin-1 and cell membrane, molecular dynamics simulations were performed. The results showed that the binding of lachnospirin-1 to the bacterial cell membrane was increasingly tight during the simulations, while no binding was observed between lachnospirin-1 and mammalian cell membranes (Figures 3A and S8). Analyses of binding energy also revealed that lachnospirin-1 had a stronger binding affinity to the bacterial cell membrane than mammalian cell membrane (Figure 3(B)). Further analysis indicates that lachnospirin-1 mainly binds to the bacterial cell membrane through hydrogen bonding interactions (Figure 3(C)). Competitive inhibition experiments conducted on cell membrane components POPC, POPG, and CL indicated that lachnospirin-1 mainly acts on the cell membrane components of POPG and CL (Figure 3(E)). Meanwhile, NPN tracing demonstrated that lachnospirin-1 can increase the outer membrane permeability (Figure 3(D)).

LPS is a unique component of the outer wall of Gram-negative bacteria and the core component of endotoxin, playing a crucial role in bacterial virulence. Competitive inhibition experiments using exogenous LPS showed that lachnospirin-1 exhibited affinity to LPS (Figure S9). Molecular docking further showed the binding between LPS and lachnospirin-1, which indicated hydrogen bonding, salt bridges, hydrophobic interactions, electrostatic interactions, and C-H bonding were the main interactions (Figure 3(F)). The ITC further confirmed the direct specific binding between lachnospirin-1 and LPS with the Kd value of 3.8 µM (Figure 3(G)). In summary, the AMP lachnospirin-1 can bind to the LPS of *A. baumannii*, as well as disrupting its cell membranes by targeting POPG and CL.

### Lachnospirin-1 induces oxidative stress leading to ROS accumulation

Next, we explore an additional mechanism of lachnospirin-1. The intracellular ATP was decreased after being treated with lachnospirin-1 in a dose-dependent manner (Figure 4(A)). However, the ROS level was largely increased in the presence of lachnospirin-1 as determined by the DCFH-DA probe (Figure 4(B,D)). To further explore the specific component contribution to the enhanced ROS, probes of HKperox-2, HKSOX-1, and HKOH-1 r were used for the detection of H_2_O_2_, •O^2-^, •OH, respectively. And it was found that the H_2_O_2_ was the main component of the increased ROS induced by lachnospirin-1 (Figure 4(E)). While there were no significant changes in the component of •OH (Figure 4(F)) and •O^2-^ (Figure 4(G)). As expected, the bactericidal ability of lachnospirin-1 against *A. baumannii* was impaired, when added with the ROS scavenger GSH and thiourea (Figure 4(C)). In conclusion, we found that inducing oxidative stress to stimulate ROS accumulation could be an additional antimicrobial mechanism by lachnospirin-1 (Figure 4(H)).

### Effective *in* *vivo* antibacterial efficacy of lachnospirin-1

We established both a mouse cutaneous abscess model and a wound infection model to investigate the bactericidal effects of lachnospirin-1 *in vivo*. In the abscess model, we observed that the abscesses in the mice administered with lachnospirin-1 exhibited reduced infection size with lower bacterial load than that in the control group (Figure 5(A)). In Figure 5(B), through the plate colony counting method, we could intuitively observe that lachnospirin-1 can effectively reduce the bacterial load in the abscess. In accordance, H&E staining also showed a decrease in inflammatory infiltration after the treatment with lachnospirin-1 (Figure 5(C)). Similarly, as shown in Figure 5(D), the wound infection model indicated that the bacterial load in the lachnospirin-1-treated group was significantly decreased than that in the control group. The levels of inflammatory cytokines in the lachnospirin-1-treated mice were also significantly diminished (Figure 5(E)). Meanwhile, H&E staining revealed an obvious reduction in inflammatory infiltration after the lachnospirin-1 administration (Figure 5(F)).

### Safety profile of lachnospirin-1

The hemolysis rate of lachnospirin-1 induced in human RBCs remains extremely low even at concentrations up to 256 µM (Figure 2). To verify the *in vivo* toxicity of lachnospirin-1, we intraperitoneally injected normal healthy mice with 20 mg/kg lachnospirin-1, and evaluated their organic functions as well as pathological changes. Comparison of the liver and kidney function biomarkers between the groups revealed no statistically significant differences (Figure 6(A)). Meanwhile, the H&E staining of organs, including the heart, liver, spleen, lungs, and kidneys, showed no pathological abnormalities (Figure 6(B)). Thus, lachnospirin-1 has extremely low *in vitro* and *in vivo* toxicity with high potential for clinical applications.

## Discussion

The high rates of infection and mortality associated with *A. baumannii*, compounded by its antibiotic resistance, present a major global public health challenge and significantly complicate clinical treatment [55]. Therefore, exploring new treatment strategies is urgent, and AMPs are promising potential alternatives to antibiotic therapy. In this study, we synthesized the AMP lachnospirin-1 and found that it exerts excellent antibacterial activity against MDR-AB and its biofilms both *in vitro* and *in vivo*. Lachnospirin-1 also exhibits certain bactericidal activity against other Gram-negative or Gram-positive bacteria with reliable safety. Lachnospirin-1 possesses multiple mechanisms, including disrupting bacterial cell membranes, neutralizing LPS, and inducing oxidative stress, and exhibits.

A systematic review indicated that cell wall-targeting agents are the focus of antibiotic drug development, while most AMPs targeting *A. baumannii* can act on bacterial cell membranes [56]. In this study, we determined the effect of lachnospirin-1 on bacterial cell membranes through experiments, such as fluorescent staining with probes (including PI, SYTOX GREEN, and DiSC35) and molecular docking. We found that lachnospirin-1 can specifically bind to LPS on the outer membrane of *A. baumannii* and disrupt the bacterial membrane structure. In addition, lachnospirin-1 can induce oxidative stress, leading to an increase in ROS (Figure 4(B)). In conclusion, these multiple mechanisms of lachnospirin-1 can effectively reduce the development of drug resistance. This further confirms the favorable clinical application prospects of lachnospirin-1 demonstrated in this study.

Currently, the exploration of novel AMPs is a research hotspot in the field of drug-resistant bacterial infections. AMPs hold enormous therapeutic potential in combating superbugs, as they can exhibit potent bactericidal activity within the micromolar range, along with rapid killing efficacy and low propensity for inducing drug resistance [57]. In this study, the results of LC_50_, MIC, and time–kill curves confirm that lachnospirin-1 demonstrates strong bactericidal activity and rapid killing within the micromolar range. As shown in Figure 4, its antibacterial efficacy is even slightly superior to that of clinically applied melittin. Compared with currently developed anti-*A. baumannii* AMPs (SAAP-148, LC_99.9_ = 0.8 µM [25]; Cec4, MIC = 4 µg/mL [58]), its antibacterial efficacy is equally potent. Although, colistin and polymyxin have been widely used in clinical practice due to their significant killing effect against *A. baumannii*, playing a crucial role in the treatment of *A. baumannii* infections [59,60], their application is still limited by excessive nephrotoxicity, which imposes strict requirements on dosage control [61]. In comparison, no *in vivo* toxicity was observed by lachnospirin-1 in this study.

Bacterial biofilms are widely present in nosocomial and implant-related infections, which reduces the sensitivity of bacteria to antibiotics [62]. In our study, we found that lachnospirin-1 can exert a significant killing effect against the biofilms of MDR-AB at relatively low concentrations. CLSM and crystal violet staining also demonstrated lachnospirin-1’s ability to eliminate preformed biofilms. Similarly, Rajapaksha DC et al. [63] discovered that the AMP Octopromycin can inhibit *A. baumannii* biofilm formation and quorum sensing, however, it requires a high concentration of 200 µg/mL to effectively remove biofilms. In contrast, lachnospirin-1 could exhibit antimicrobial activity against preformed biofilms at a concentration of 1 µM. Nevertheless, the mechanism by which lachnospirin-1 clears biofilms has not been thoroughly investigated in this study, and further in-depth research could be conducted in the future. Persister cells usually occur in long-term chronic infections and exhibit a certain degree of tolerance to antibiotics, making clinical treatment challenging [64]. Studies have reported that blue benzoquinone derived from scorpion venom has excellent bactericidal activity against the persister cells of *A. baumannii*, with an extremely low possibility of inducing drug resistance [65]. However, lachnospirin-1 not only exerts highly effective elimination of persister bacteria of MDR-AB but also shows good killing efficacy against persister bacteria of other Gram-negative bacteria, such as *P. aeruginosa* and *K. pneumoniae*. And its multiple bactericidal mechanisms allow the prediction of an extremely low likelihood of drug resistance development.

Poor stability of AMPs in physiological environments is one of the major obstacles restricting their clinical application. Most AMPs show a significant reduction or even a complete loss of antimicrobial activity in serum or physiological salt environments [66,67]. Studies suggest that this phenomenon mainly stems from two factors: first, the binding interaction with anionic serum proteins (primarily serum albumin), and second, the hydrolytic action of proteases [67]. As shown in Figure 1(H), the antimicrobial activity of lachnospirin-1 is notably inhibited in the presence of Na^+^, Mg^2+^, and Ca^2+^. This is consistent with the phenomenon observed in existing studies [68–70]. Compared with AMPs currently under development (e.g. F3FT, and N3NT [50]), it has slightly lower stability, thus requiring subsequent structural optimization to enhance its stability. However, we discovered that the antimicrobial activity of lachnospirin-1 is enhanced in the presence of NH_4_^+^. We hypothesize that this may be related to NH_4_^+^ altering the local microenvironment. Meanwhile, this finding also inspires us to add ammonium-containing components (such as ammonium chloride and ammonium sulfate) to pharmaceutical formulations to enhance antimicrobial efficacy. But systemic administration is likely to cause hyperammonemia due to the addition of NH_4_^+^; therefore, it is more suitable for topic administration in clinical practice.

In conclusion, the AMP lachnospirin-1 exhibits significant bactericidal activity against CR-AB and can effectively eliminate its biofilms and persister bacteria. Lachnospirin-1 exerts its effects through multiple mechanisms of disrupting cell membranes, targeting lipopolysaccharides, and inducing oxidative stress, thus having a low likelihood of triggering drug resistance and great potential for clinical applications. Additionally, lachnospirin-1 satisfies toxicity profile and *in vivo* bioactivity. However, the study on the stability of lachnospirin-1 still have limitations. It only detects the stability of lachnospirin-1 in serum and inorganic salts in a single way, while ignoring the complexity of the *in vivo* environment and failing to involve the influence of factors, such as pH and temperature. Although we identified the synergistic effect of NH_4_^+^ on the activity of lachnospirin-1 and proposed relevant hypotheses, we failed to investigate the underlying mechanism due to technical limitations. Meanwhile, since this study is still in the preliminary stage, in-depth investigations into resistance screening assays and toxicity tests have not been conducted, which will be progressively carried out in subsequent research. A substantial gap still exists for lachnospirin-1’s translation into clinical practice. Despite mouse models and humans belonging to the same class of mammals, further experiments using large mammals must be conducted prior to the initiation of clinical trials. Collectively, these results indicate that lachnospirin-1 serves as a potential lead compound against CRAB infections, warranting further structural optimization in subsequent studies.

## Supplementary Material

Revised_Table1_clean.docx

Author Checklist.pdf

Revised_Supplementary_Figures_clean.docx

Revised_Figure_Legends clean.docx

## Data Availability

All datasets generated for this study are included in this article and the supplementary files. The data that support the findings of this study are available in zenodo at https://doi.org/10.5281/zenodo.18457753 [71].

## Animal experiments statement

All animal experiments in this study were conducted in compliance with the ARRIVE guidelines to ensure ethical and transparent reporting. The grouping was conducted by Guanqing Huang, who did not participate in the subsequent experiments. Mengna Li carried out the subsequent experiments without knowing the grouping information, while Pengfei She was responsible for data analysis and was aware of the grouping details.
