# Supplementary material for: Antibacterial efficacy and mechanism of the novel antimicrobial peptide lachnospirin-1 against Acinetobacter baumannii
Source: Virulence. 2026 Mar 16;17(1):2646808. doi: 10.1080/21505594.2026.2646808 (PMC13007424; doi:10.1080/21505594.2026.2646808)
Supplement: Revised_Table1_clean.docx [file KVIR_A_2646808_SM3302.docx]

**Table1. Antibacterial susceptibility of Lachnospirin-1 against pathogens**

|  | **LC_50_(µM)** | **MIC(µM)** | **MIC(µg/ml)** | |
| --- | --- | --- | --- | --- |
| **Strain** | Lachnospirin-1 | Lachnospirin-1 | PolyB | VAN |
| *A. baumannii* | | | | |
| ATCC 19606 | 1.8 | 2 | 2 | — |
| ATCC 1195 | — | 2 | 2 | — |
| AB 1069*^a^ | 0.7 | 2 | 4 | — |
| AB 1280*^a^ | 0.6 | 8 | 4 | — |
| AB 1085 | 0.1 | 2 | 2 | — |
| AB 1 | — | 4 | 4 | — |
| AB 2 | — | 4 | 4 | — |
| AB 3 | — | 2 | 4 | — |
| AB 4 | — | 32 | 4 | — |
| AB 5 | — | 4 | 2 | — |
| *K. pneumoniae* | | | | |
| ATCC 700603 | ＞4 | ＞64 | 2 | — |
| ATCC 13883 | — | 8 | 2 | — |
| *E. faecium* | |  |  |  |
| VSE1 | ＞4 | 4 | — | 2 |
| *P. aeruginosa* | | | | |
| PAO1 | 0.5 | 4 | 2 | — |
| PA 47 | — | 32 | 2 | — |
| *E. coli* | | | | |
| ATCC 25922 | ＞4 | 4 | 4 | — |
| *S. aureus* | | | | |
| ATCC 43300 | — | ＞64 | — | 1 |
| SAJ1 | ＞4 | 1 | — | 8 |

*, MRD-AB; ^a^, CRAB; —, no date.
