# Supplementary material for: Antibacterial efficacy and mechanism of the novel antimicrobial peptide lachnospirin-1 against Acinetobacter baumannii
Source: Virulence. 2026 Mar 16;17(1):2646808. doi: 10.1080/21505594.2026.2646808 (PMC13007424; doi:10.1080/21505594.2026.2646808)
Supplement: Revised_Supplementary_Figures_clean.docx [file KVIR_A_2646808_SM3300.docx]

**Supplementary Information**

**Antibacterial Efficacy and Mechanism of the Novel Antimicrobial Peptide Lachnospirin-1 Against *Acinetobacter baumannii***

**Pengfei She^1#^, Mengna Li^2,4#^, Yiqing Liu^2,4^, Guanqing Huang^2,4^, Shaowei Guo^3,4^, Dan Xiao^3,4^, Yelan Hong^3,4^, Lihua Lu^3,4^, Yong Wu^2,3,4*^**

*1.Department of Laboratory Medicine, the Third Xiangya Hosipital, Central South University, Changsha, 410003, China*

*2.Department of Laboratory Medicine, Xiangya Hosipital, Central South University, Changsha, 410008, China*

*3.Department of Laboratory Medicine, Postgraduate Cooperative Training Base of Changsha First Hospital, Hengyang Medical College, University of South China, Changsha, 410005, China*

*4.Department of Laboratory Medicine, Changsha First Hospital (Affiliated Changsha Hospital of Xiangya School of Medicine,Central South University), Changsha, 410005, China*

***Corresponding author:**

Yong Wu, Department of Laboratory Medicine, Changsha First Hospital (Affiliated Changsha Hospital of Xiangya School of Medicine, Central South University), Changsha, 410005, China. Email:wuyong_xy@163.com. Address: No.311 Yingpan Rd. Changsha, China，410005.

**Table S1.** Antimicrobial susceptibility testing of multidrug-resistant strains

| **Common Clinical Antibiotics** | **AB 1069** | **AB 1280** | **AB 1085** |
| --- | --- | --- | --- |
| Ticarcillin/Clavulanic Acid | R | R | S |
| Piperacillin/Tazobactam | R | R | S |
| Ceftazidime | R | R | S |
| Cefoperazone/Sulbactam | R | R | S |
| Cefepime | R | R | S |
| Imipenem | R | R | S |
| Meropenem | R | S | S |
| Tobramycin | R | R | S |
| Ciprofloxacin | R | R | S |
| Levofloxacin | R | R | S |
| Doxycycline | R | R | S |
| Minocycline | I | I | S |
| Tigecycline | I | S | S |
| Colistin | S | S | S |
| Trimethoprim/Sulfamethoxazole | R | S | S |

R, resistant; I, intermediate; S, susceptible.

**Table S2. Comparison of antibacterial activities of three AMP**s

|  | **MIC (µM)** | | |
| --- | --- | --- | --- |
| **Strains** | Lachnospirin-1 | Enterococcin-1 | Ampspherin-4 |
| ATCC19606 | 2 | 4 | ＞64 |
| AB1069 | 2 | 4 | ＞64 |
| AB1085 | 2 | 64 | ＞64 |


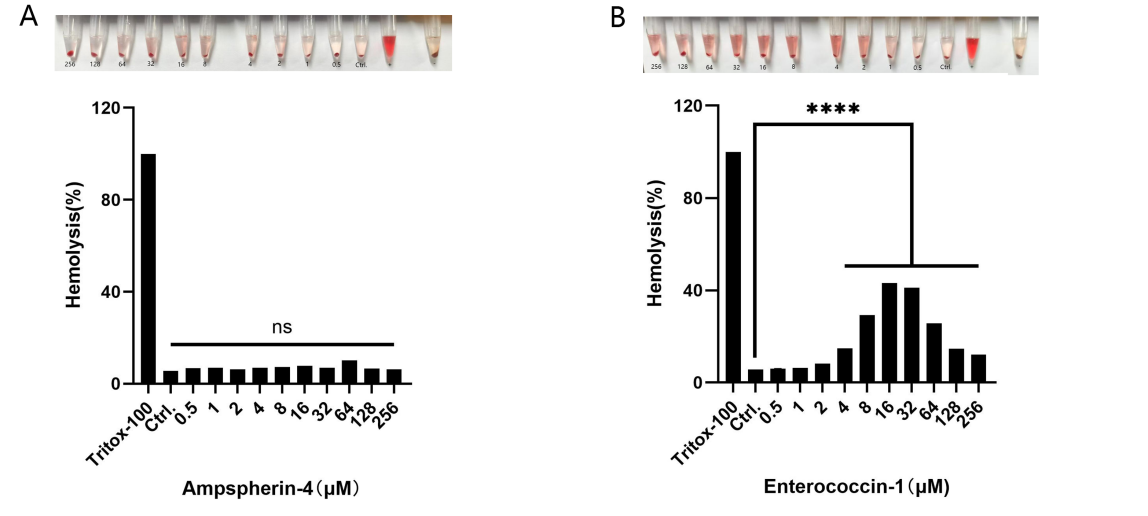


**Fig.S1 Antibacterial and hemolysis activity of antimicrobial peptides.** (A) The red blood cell toxicity of the antimicrobial peptide Ampspherin-4. (B) The red blood cell toxicity of Enterococcin-1. ****, *P* < 0.0001; ns, no statistical significance.


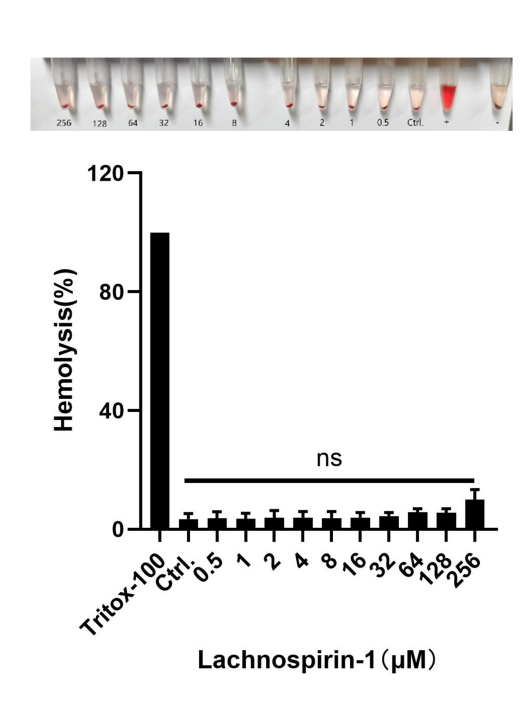


**Fig.S2 Hemolytic activity of Lachnospirin-1 on red blood cells.** ns, no statistical significance.


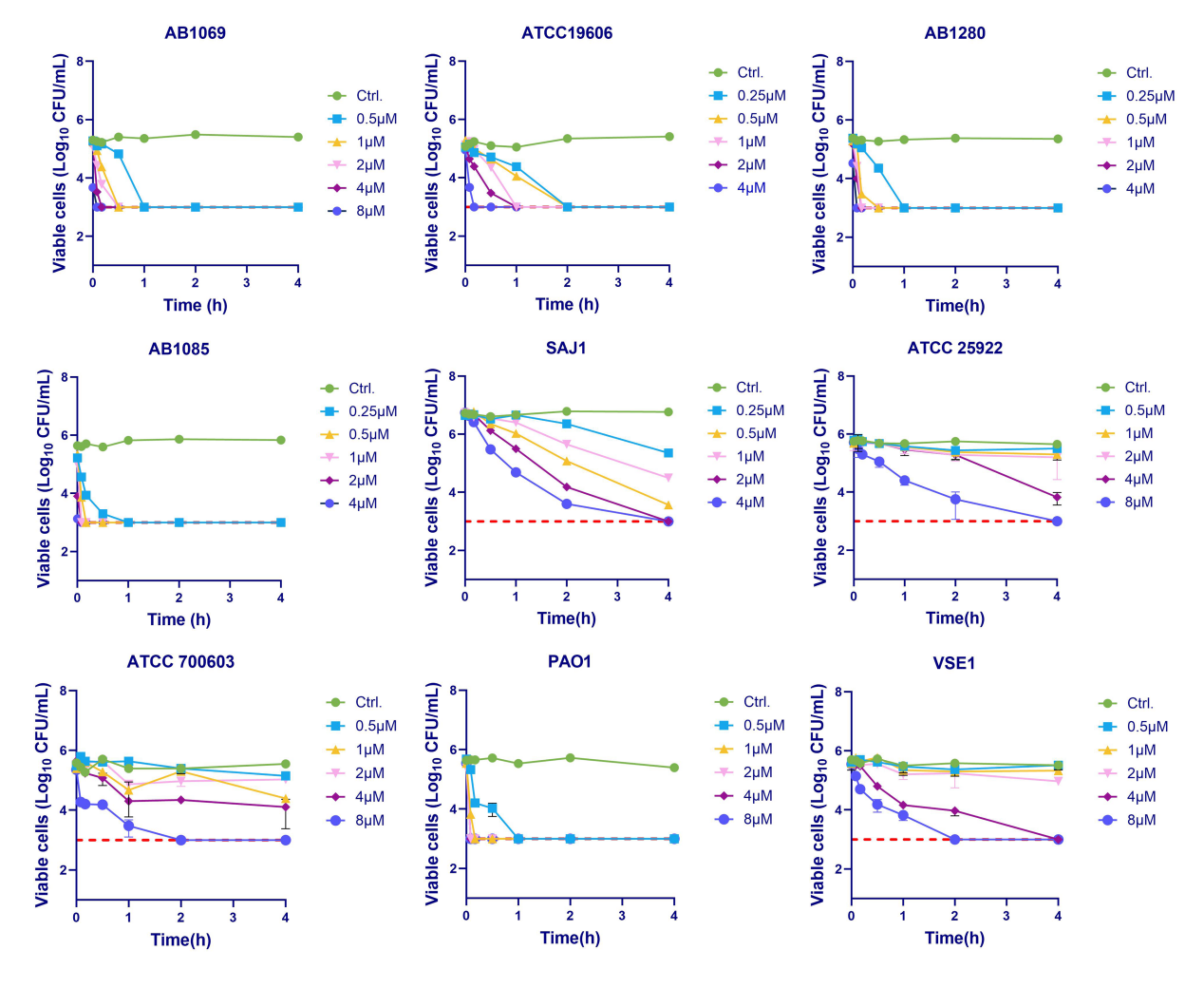


**Fig.S3 Short-term antibacterial efficacy of Lachnospirin-1 against pathogens.** Antibacterial activity of Lachnospirin-1 against *A. baumannii*, *S. aureus*, *E. coli*, *K. pneumoniae*, *P. aeruginosa*, and Enterococcus spp. The red dashed line represents the limit of detection. *A. baumannii*: AB 1069, ATCC 19606, AB 1280, AB 1085; *S. aureus*: SAJ1;*E. coli*: ATCC 25922; *K. pneumoniae*: ATCC 700603; *P. aeruginosa*: PAO1; Enterococcus spp.: VSE1.


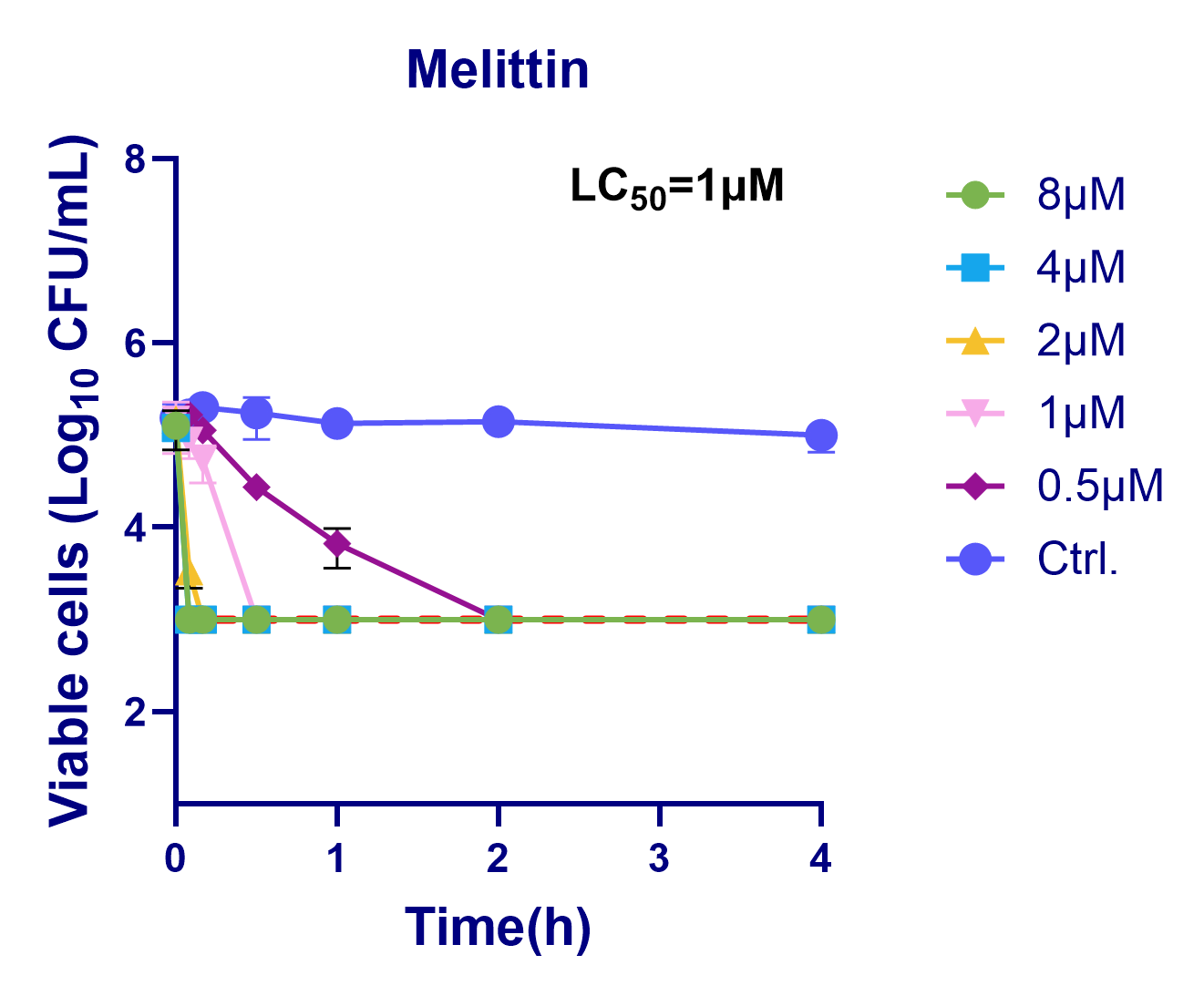


**Fig.S4 The antibacterial activity of melittin against AB1069.** The red dashed line represents the detection line.


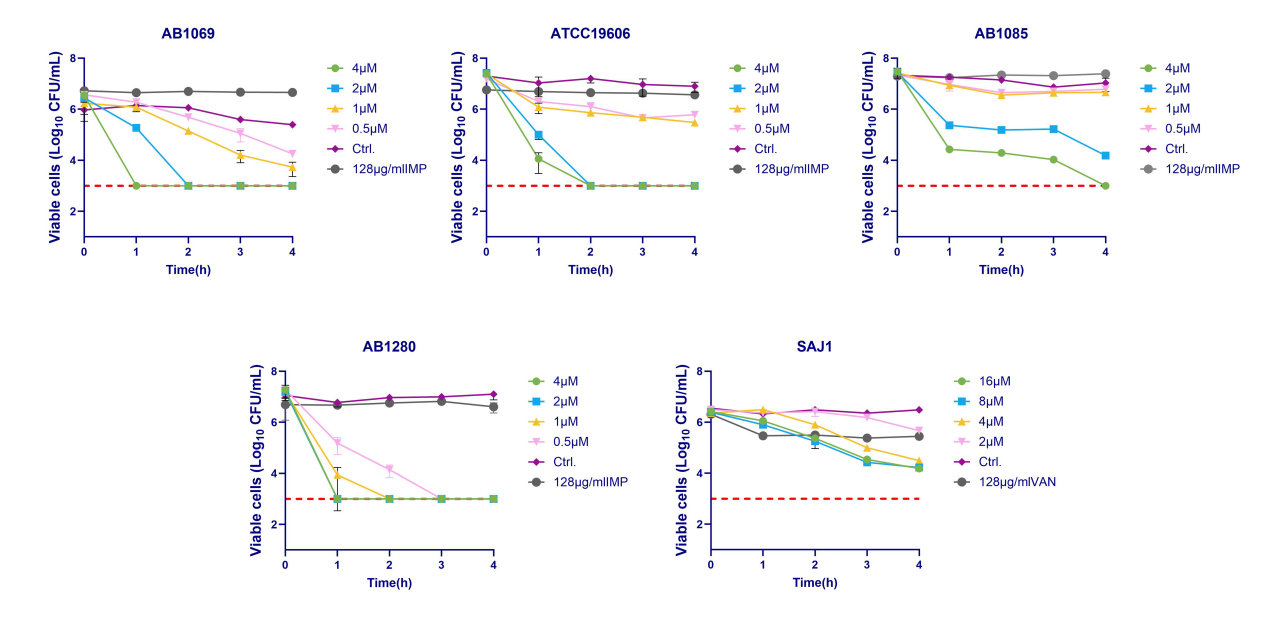


**Fig.S5 Antibacterial efficacy of Lachnospirin-1 against persister cells.** Time-kill assay results of Lachnospirin-1 against persister cells of *A. baumannii* and *S. aureus*. The red dashed line represents the detection line. *A. baumannii*:AB1069, AB1280, AB1085, ATCC19606; *S. aureus*:SAJ1.IMP, imipenem; VAN, vancomycin.


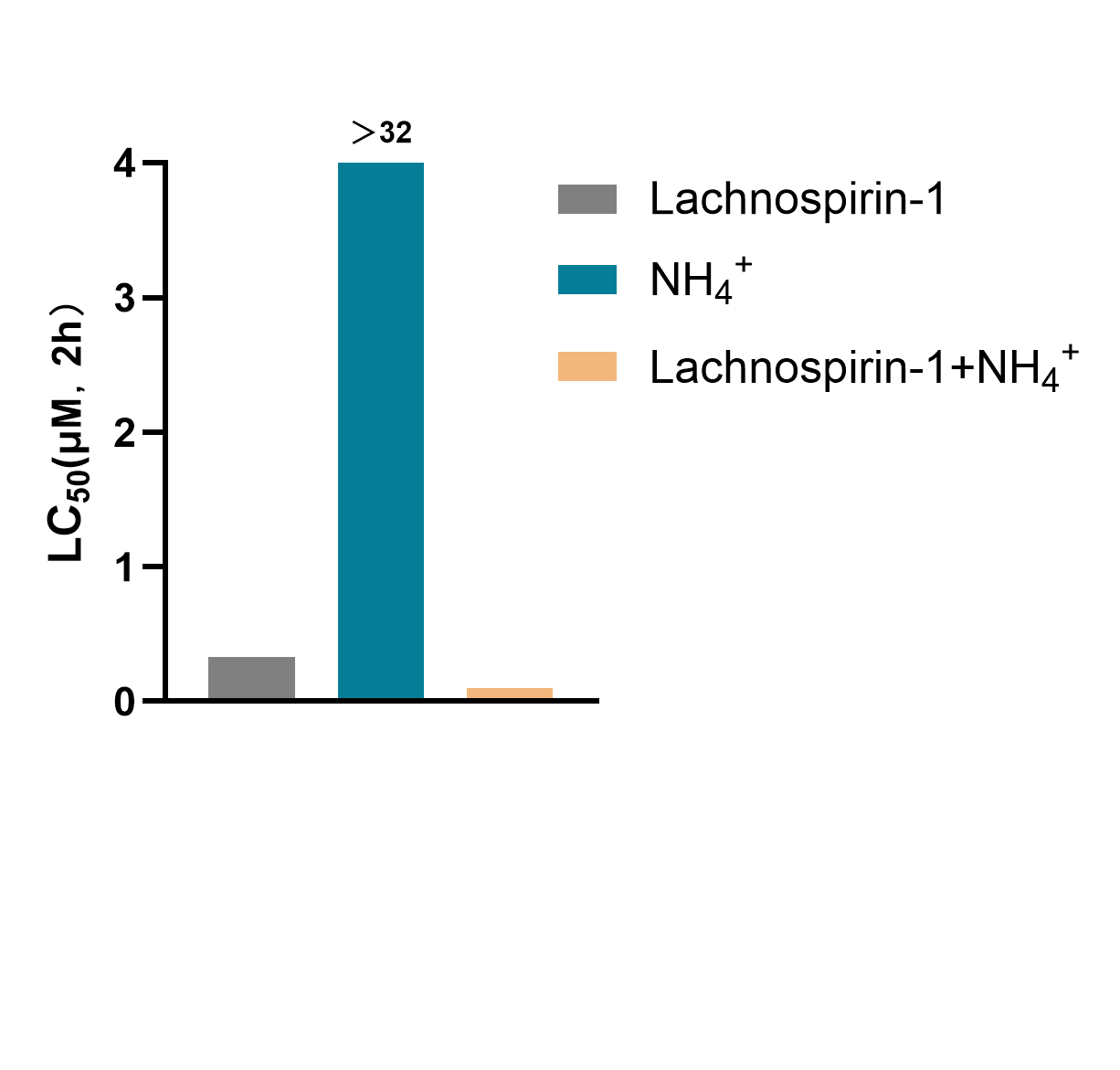


**Fig.S6 Synergistic effect between NH_4_^+^ and Lachnospirin-1.**


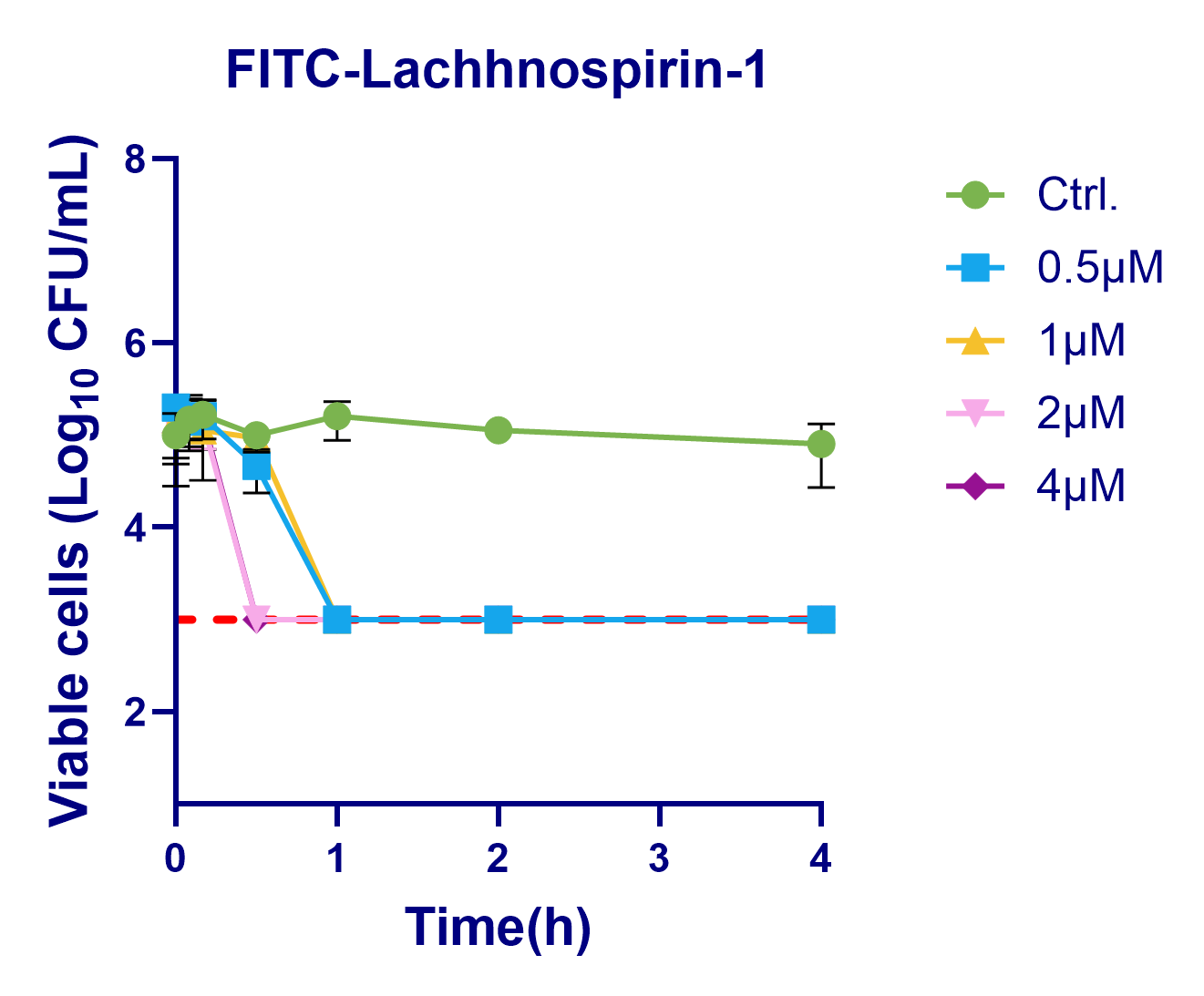


**Fig.S7 Antibacterial activity of FITC-Lachnospirin-1.** The red dashed line represents the limit of detection.


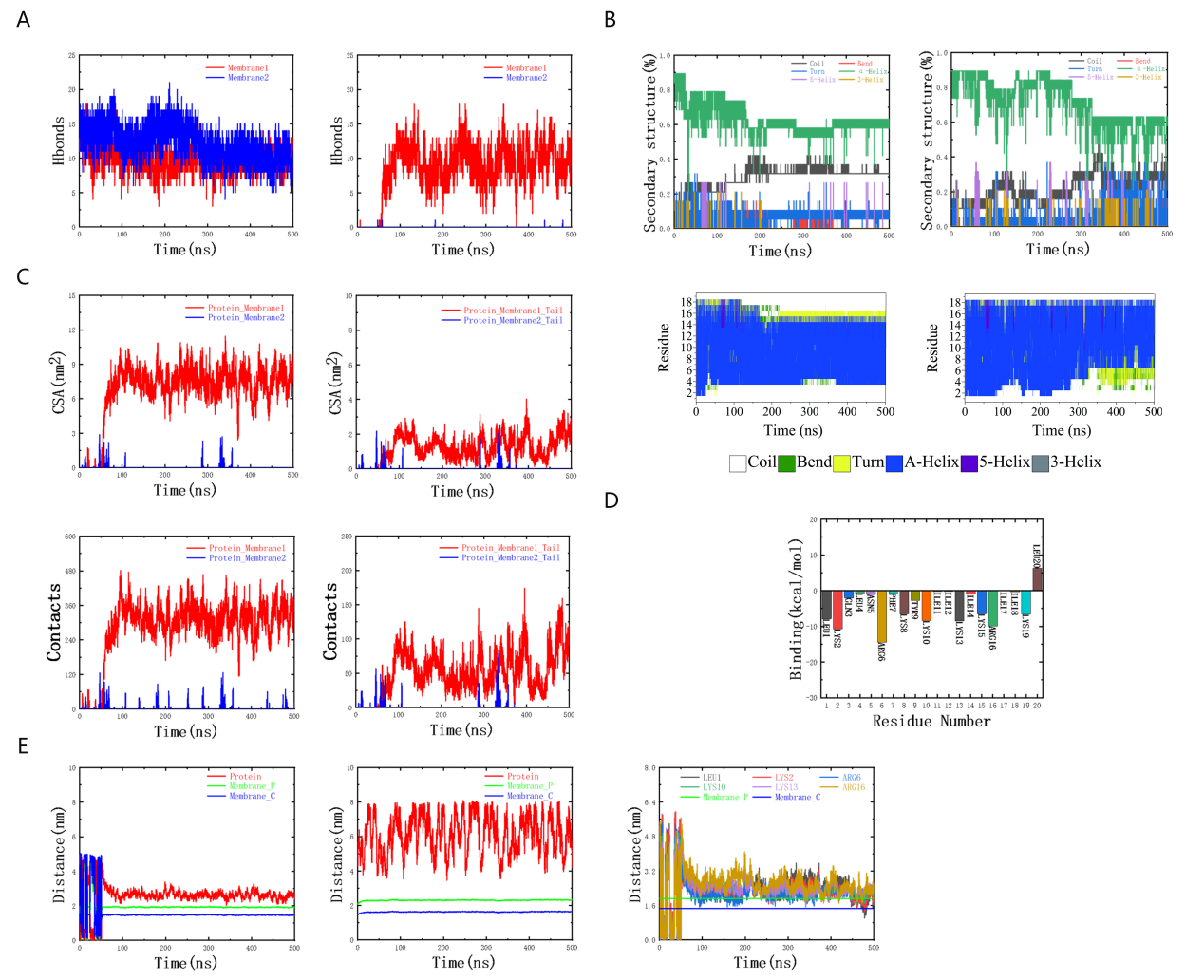


**Fig.S8 Detailed analysis of the molecular dynamic simulations.** (A) Hydrogen bond analysis during the molecular dynamics process. The left figure shows the change in the number of intramolecular hydrogen bonds of the AMP, while the right figure shows the number of hydrogen bonds between the antimicrobial peptide and the cell membrane. (B) Secondary structure analysis of the antimicrobial peptide during the molecular dynamics process. The upper left figure presents the content change of each secondary structure of the AMP in the mixed cell membrane system of DOPC and DOPG; the upper right figure shows the content change of each secondary structure of the AMP in the mixed cell membrane system of POPC and Cholesterol; the lower left figure displays the Dictionary of Secondary Structure of Proteins (DSSP) for the amino acid residues of the antimicrobial peptide in the DOPC-DOPG mixed cell membrane system; the lower right figure shows the DSSP for the amino acid residues of the antimicrobial peptide in the POPC-Cholesterol mixed cell membrane system.(C) Contact analysis between the antimicrobial peptide and the cell membrane (including the membrane tail) during the molecular dynamics process: The upper left figure indicates the contact surface area between the antimicrobial peptide and the DOPC-DOPG mixed cell membrane; the upper right figure shows the contact surface area between the antimicrobial peptide and the hydrophobic tail of the DOPC-DOPG mixed cell membrane; the lower left figure presents the number of contact atoms between the antimicrobial peptide and the POPC-Cholesterol mixed cell membrane; the lower right figure displays the number of contact atoms between the antimicrobial peptide and the hydrophobic tail of the POPC-Cholesterol mixed cell membrane. (D) Contribution of amino acid residues of the antimicrobial peptide to the binding free energy in the DOPC-DOPG mixed cell membrane system. (E) Centroid distance analysis during the molecular dynamics process: The left figure shows the centroid distance between the antimicrobial peptide and the DOPC-DOPG mixed cell membrane; the middle figure presents the centroid distance between the antimicrobial peptide and the POPC-Cholesterol mixed cell membrane; the right figure displays the centroid distances between several key binding amino acids and the DOPC-DOPG mixed cell membrane. "Protein" refers to the centroid distance between the antimicrobial peptide and the cell membrane; "Membrane_P" denotes the plane where the phosphorus atoms of phospholipid molecules are located; "Membrane_C" represents the plane where the carbonyl carbon atoms of phospholipid molecules are located. "Membrane1" stands for the DOPC-DOPG mixed cell membrane system, and "Membrane2" refers to the POPC-Cholesterol mixed cell membrane system.


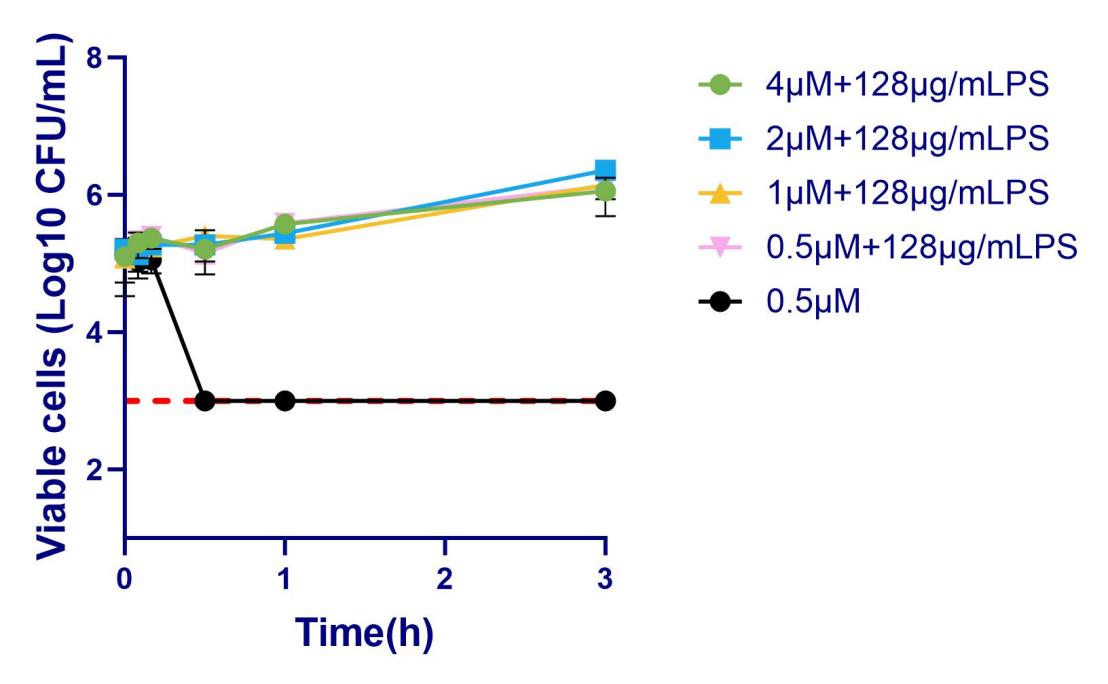


**Fig.S9 Competitive inhibition experiment of lachnospirin-1 with LPS.** The red dashed line represents the limit of detection.
