# Supplementary material for: Antibacterial efficacy and mechanism of the novel antimicrobial peptide lachnospirin-1 against Acinetobacter baumannii
Source: Virulence. 2026 Mar 16;17(1):2646808. doi: 10.1080/21505594.2026.2646808 (PMC13007424; doi:10.1080/21505594.2026.2646808)
Supplement: Revised_Figure_Legends clean.docx [file KVIR_A_2646808_SM3299.docx]

Figure Legends


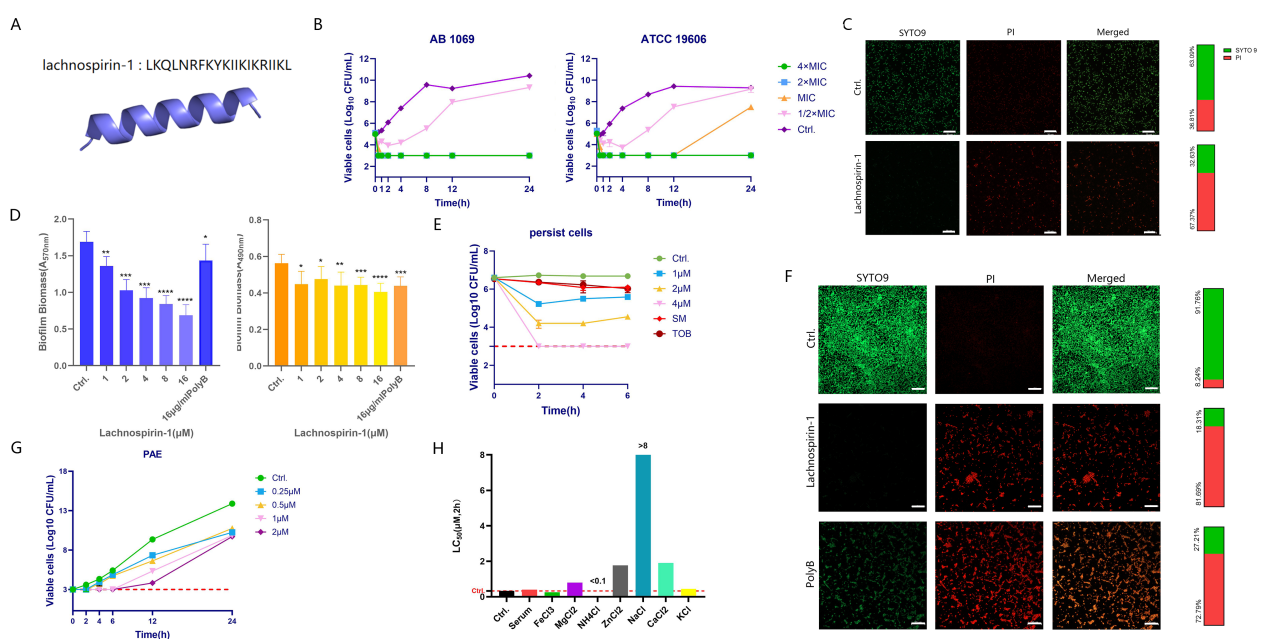


**Fig.1 *In v-itro* antibacterial effects of lachnospirin-1.** (A) Structure and sequence of lachnospirin-1. (B) Bactericidal activity of lachnospirin-1 against *A. baumannii* determined by CFU count. (C) Live/dead bacterial staining. Scale:20 µm. (D) Quantification of biofilms by CV and XTT reduction assay. (E) Antibacterial activity of lachnospirin-1 against persister bacteria. Tob: Tobramycin; SM: Streptomycin. (F) Anti-biofilm effect under confocal microscopy. Scale:20 µm. (G) Post-antibiotic effect of lachnospirin-1. (H) Stability of lachnospirin-1 in serum and inorganic salts.


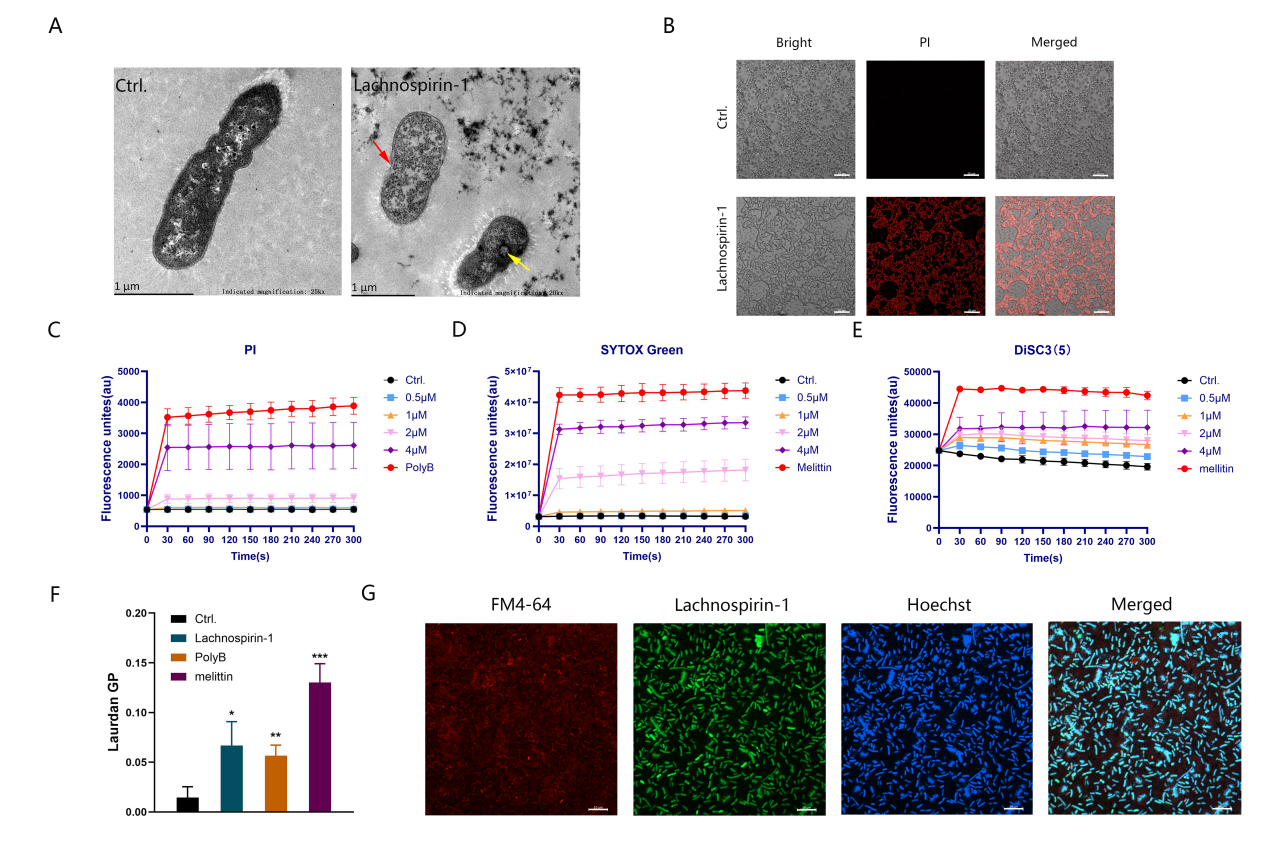


**Fig.2 Lachnospirin-1 acts on the cell membrane of AB1069.**(A) Observation of the effect of lachnospirin-1 on the cell membrane under TEM. Scale:1 µm. Red arrows indicate cell membrane rupture and content leakage; yellow arrows indicate the appearance of mesosomes. (B) Observation of PI staining fluorescence intensity under CLSM. Scale:20 µm. (C) Quantitative analysis of PI staining. (D) Quantitative analysis of SYTOX GREEN staining. (E) Detection of membrane potential using DiSC3(5) probe. (F) Detection of membrane fluidity by Luardan staining. (G) Fluorescence labeling of FITC-lachnospirin-1 observed under CLSM, showing its localization in the cytoplasm of *A. baumannii*. Green fluorescence represents FITC-lachnospirin-1; red fluorescence represents the membrane stain FM4-64; blue fluorescence represents the DNA-stained Hoechst 33342. Scale:10 µm.


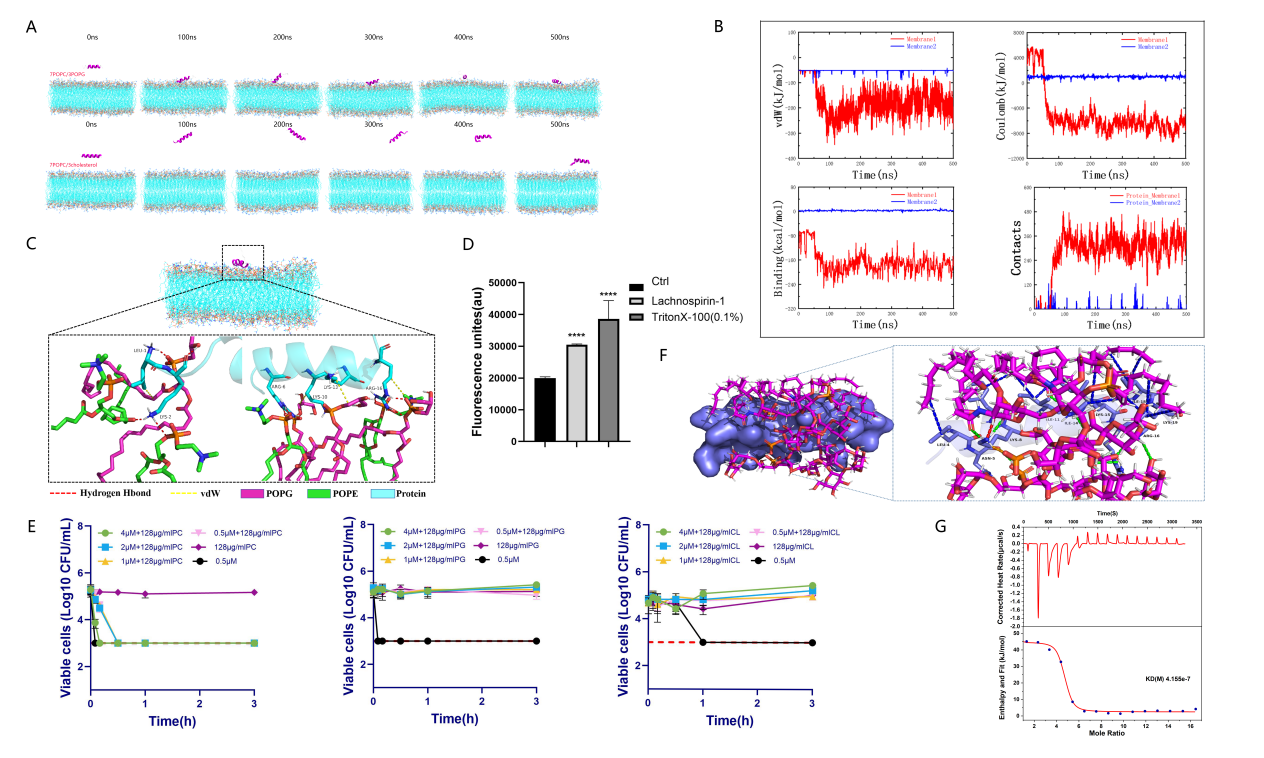


**Fig.3 Lachnospirin-1 can specifically bind to LPS to target bacterial cell membrane..** (A) Molecular dynamics simulation diagrams of lachnospirin-1 interacting with cell membranes. The upper diagram shows bacterial membranes, and the lower diagram shows mammalian membranes. From left to right: simulation initiation, membrane attachment, membrane penetration, and the equilibrium state of lachnospirin-1 interacting with the cell membrane. (B) Energy analysis during molecular dynamics simulation. The upper left panel shows the van der Waals interaction energy between the antimicrobial peptide and the cell membrane; the upper right panel shows the electrostatic interaction energy (Coulomb) between the antimicrobial peptide and the cell membrane; the lower left panel shows the binding free energy between the antimicrobial peptide and the cell membrane; the lower right panel shows the binding free energy contribution of amino acid residues of the antimicrobial peptide in the mixed cell membrane system of DOPC and DOPG. Membrane1 refers to the mixed cell membrane system of DOPC and DOPG; Membrane2 refers to the mixed cell membrane system of POPC and Cholesterol. (C) Details of the interaction between the AMP and the mixed cell membrane of DOPC and DOPG during molecular dynamics simulation. (D) Detection of bacterial cell membrane permeability after lachnospirin-1 treatment using NPN probe. (E) Competitive inhibition experiment of lachnospirin-1 with cell membrane components POPC, POPG, and CL. (F) Schematic diagram of the interaction structure between LPS and lachnospirin-1. Blue sticks represent lachnospirin-1 residues; magenta sticks represent LPS molecules; green dashed lines represent hydrogen bond interactions; light green dashed lines represent C-H bond interactions; red dashed lines represent electrostatic interactions; orange dashed lines represent salt bridge interactions; blue dashed lines represent hydrophobic interactions. (G) Detection of the binding affinity between lachnospirin-1 and LPS by ITC.

.


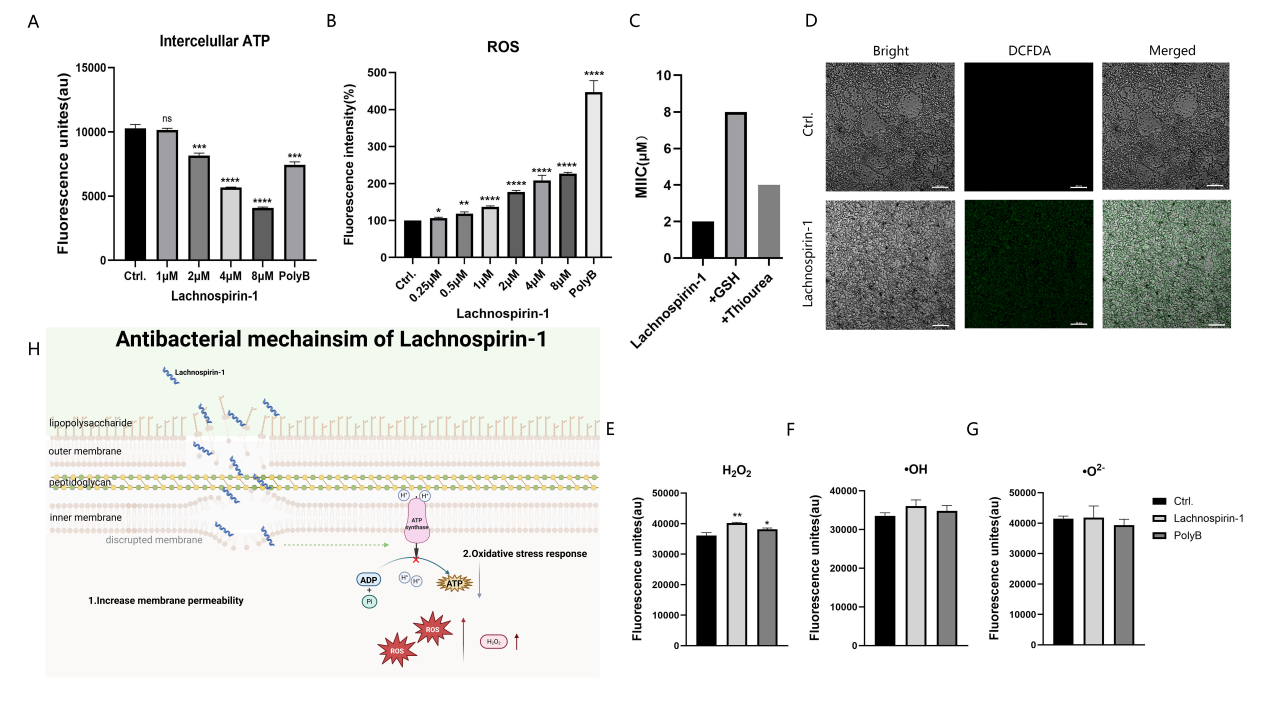


**Fig.4 Lachnospirin-1 induces oxidative stress and stimulates the accumulation of ROS.** (A) Quantitative results of intracellular ATP after treatment with lachnospirin-1. (B) Detection of intracellular ROS levels using DCFDA probe. (C) Effect of reduced GSH and thiourea on the antibacterial activity of lachnospirin-1.(D) Observation of DCF fluorescence intensity after lachnospirin-1 treatment under confocal microscopy. Scale:20 µm. (E-G) Determination of ROS component of H_2_O_2_ (E), •OH (F), •O^2-^ (G) by probes HKperox-2, HKOH-1r, and HKSOX-1, respectively. (H) Dual antibacterial mechanisms of lachnospirin-1. ADP, adenosine diphosphate; ATP, adenosine triphosphate; ROS, reactive oxygen species.*, *P* < 0.05; **, *P* < 0.01; ***, *P* < 0.001; ****, *P* < 0.0001; ns, no statistical significance.


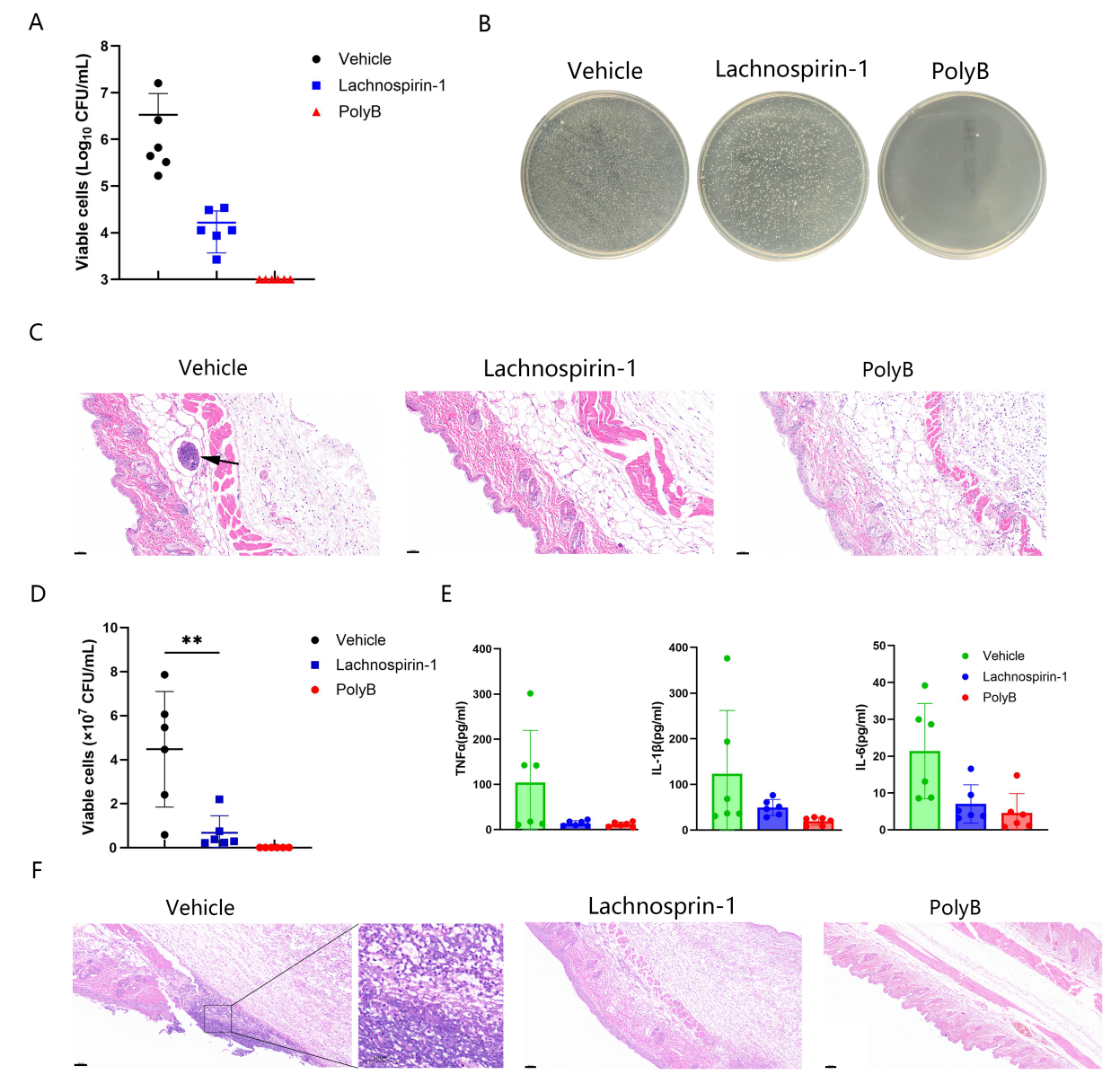


**Fig.5 Antibacterial effects of lachnospirin-1 in mouse models.** (A) Bacterial load in the skin abscess model after lachnospirin-1 treatment. (B) Observing the antibacterial effect of lachnospirin-1 via plate colony counting. (C) H&E staining of the abscess area after lachnospirin-1 treatment. Black arrows indicate abscesses. Scale:50 µm. (D) Viable bacterial loads in wounds. (E) Inflammatory factor levels quantification among groups. (F) H&E staining of the infected wounds. Scale:100 µm. The enlarged part shows the inflammatory infiltration area, scale:50 µm.**, *P* < 0.01.


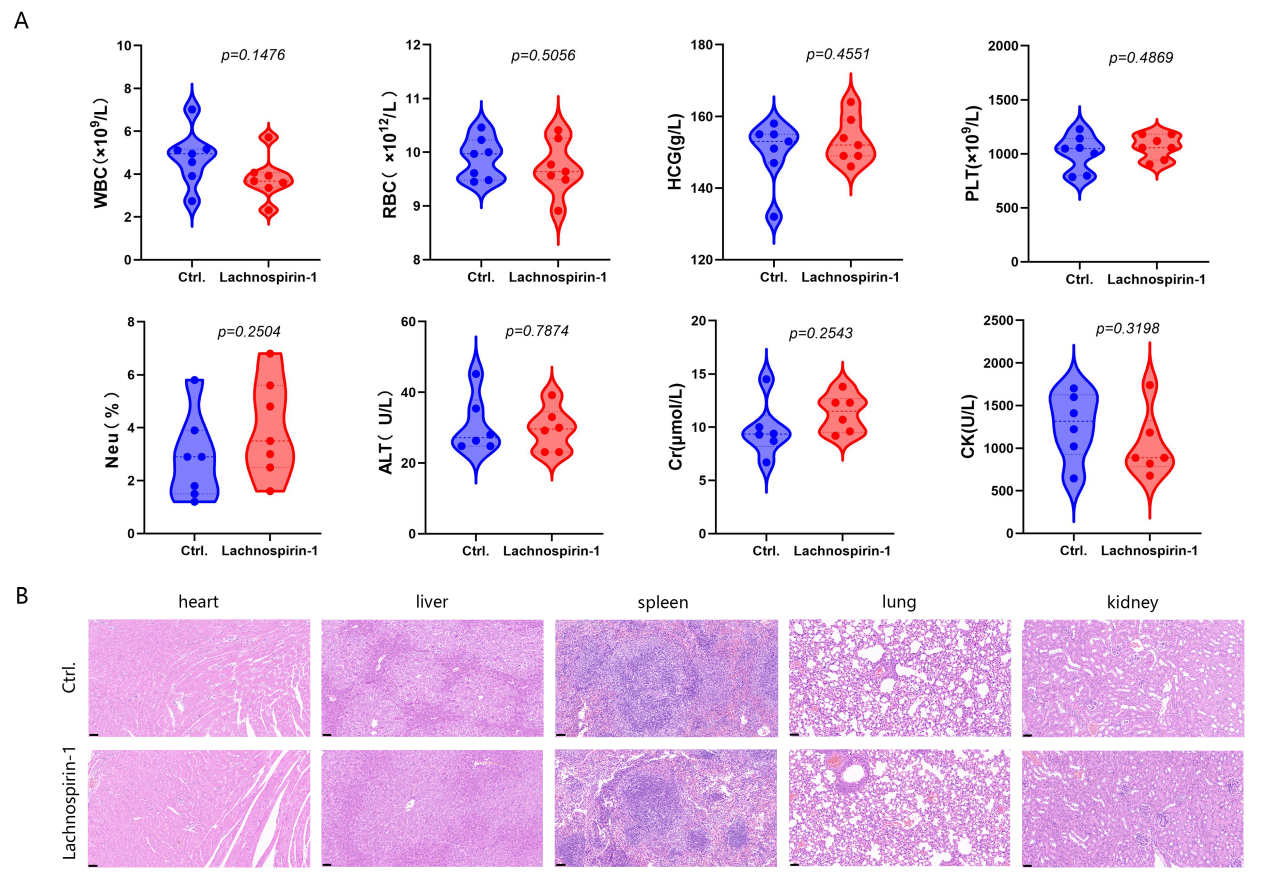


**Fig.6 *In vivo* toxicity of lachnospirin-1. (**A) Comparison of the levels of liver and kidney function markers between the two groups. (B) H&E staining of main organs.scale:50 µm.
